# Open Porous Microenvironment-regulatory Microspheres Loaded with Curcumin@BSA NPs/BMSCs for Diabetic Wound Treatment

**DOI:** 10.7150/thno.120285

**Published:** 2026-02-11

**Authors:** Zhe Liu, Qinzhou Zheng, Dong Zhou, Anqi Lin, Lan Xiao, Haifeng Liu, Keqin Ji, Huifen Qiang, Xinxin Sui, Yulin Li, Yan Wu, Jie Gao, Lan Liao, Xiaohuan Yuan

**Affiliations:** 1 School of Stomatology, Jiangxi Medical College, Jiangxi Province Key Laboratory of Oral Biomedicine, Jiangxi Province Clinical Research Center for Oral Diseases, Nanchang University, Nanchang 330006, China.; 2 College of Life Science, Mudanjiang Medical University, Mudanjiang 157011, China.; 3 Engineering Research Centre for Biomedical Materials of Ministry of Education, Frontiers Science Center for Materiobiology and Dynamic Chemistry, School of Materials Science and Engineering, East China University of Science and Technology, Shanghai 200237, China.; 4 The First Affiliated Hospital, Jiangxi Medical College, Nanchang University, Nanchang 330006, China.; 5 Changhai Clinical Research Unit, Shanghai Changhai Hospital, Naval Medical University, Shanghai 200433, China.; 6 Shanghai Key Laboratory of Nautical Medicine and Translation of Drugs and Medical Devices, Shanghai 200433, China.; 7 School of Medicine and Dentistry, Griffith University, QLD 4222, Australia.; 8 Jinggangshan University, Ji'an, Jiangxi, 343009, China.

**Keywords:** Porous microspheres, Stem cell therapy, Autophagy, Angiogenesis, Wound microenvironment remodeling

## Abstract

**Background:**

The process of healing wounds in diabetic patients is intricate and is notably obstructed by a disordered wound microenvironment, characterized by chronic inflammation and elevated blood glucose. The combination of stem cell therapy and drug treatment is seen as a promising application in future. However, the limited proliferative capacity of stem cells and inadequate drug availability present significant challenges for achieving optimal therapeutic outcomes.

**Methods:**

In this study, open porous poly (lactic‒coglycolic acid) (PLGA) microspheres were designed and synthesized via gas-assisted volatilization microemulsion technology. These microspheres encapsulate curcumin and allow its slow release, thereby enhancing wound repair. The large pores in the microspheres provide ample support for bone marrow stem cells (BMSCs), enabling continuous drug release over a period of 35 days.

**Results:**

The sustained release of curcumin promoted stem cell proliferation and maintained stem cell activity. Additionally, it facilitates remodeling of the wound immune microenvironment. Additionally, the microspheres can activate mitochondrial autophagy in cells, effectively alleviating wound inflammation.

**Conclusions:**

The combined actions of curcumin and stem cells aid in regenerating blood vessels and revitalizing the collagen network where the injury occurred, thus improving wound healing capabilities. Consequently, integrating drugs with stem cells and microspheres holds significant potential for diabetic wound treatment.

## Introduction

Diabetic wounds present significant challenges in regenerative medicine, often arising as a complication of diabetes mellitus 1. Elevated blood glucose levels impede wound healing by disrupting cell growth, differentiation, and angiogenesis 2. The healing of wounds is heavily influenced by the local skin conditions, including temperature and cleanliness 3,4. This leads to prolonged severe infections, impaired vascularization, and delayed wound closure in patients. Furthermore, chronic inflammation, a common feature in the tissue environment of diabetic individuals, exacerbates the recovery of wounded tissue 5,6. Currently, surgical debridement is the primary treatment option for diabetic wounds. However, these wounds often prove recalcitrant to healing, with symptoms persisting or worsening over time 7,8. Thus, there is a pressing requirement to design multifunctional biomaterials that can successfully facilitate the healing process in diabetic wounds.

Currently, mesenchymal stem cells (MSCs) have potential in treating diabetic wounds 9,10, especially bone marrow-derived mesenchymal stem cells (BMSCs). A variety of cytokines and growth factors makes BMSCs help regulate local cytokine levels to support wound healing and tissue regeneration 11. These properties are essential for achieving rapid and effective healing outcomes in complex wounds by reducing inflammation, accelerating skin repair, restoring barrier function, and minimizing scar formation 12. In addition, live microorganisms called probiotics, which provide health benefits when consumed in proper amounts, have been recognized as novel treatments for the recovery of diabetic wounds. Probiotics can modulate the wound microbiome, promoting a balanced microbial environment that supports tissue repair. These active therapeutic agents offer unique advantages in modulating the wound microenvironment, promoting tissue regeneration, and enhancing overall wound healing outcomes. However, the local inflammatory environment in diabetic wounds poses a significant challenge to wound healing. Consequently, a combination therapy approach that integrates stem cell therapy with anti-inflammatory drug intervention has been adopted to enhance the healing of such complex wounds. Curcumin, a traditional anti-inflammatory and healing compound, has been identified as a valuable candidate for this combination therapy 13,14. Its ability to attenuate inflammation has been demonstrated through the inhibition of various proinflammatory cytokines 15. Moreover, curcumin facilitates wound healing by actively participating in processes such as angiogenesis and cell proliferation 16. The incorporation of curcumin into a treatment regimen is expected to mitigate the detrimental effects of the local inflammatory environment and enhance the overall healing process 17. However, the limited survival of stem cells within the body and the uncontrolled release of curcumin hinder precise therapeutic delivery and timing 18.

Microspheres have become promising carriers for stem cell attachment and drug delivery, crucial for enhancing cell growth and enabling the controlled release of therapeutic substances 19. Degradable microspheres have significant potential in the fields of drug delivery and tissue regeneration. These microspheres consist of degradable polymers like polylactic acid (PLA), polycaprolactone (PCL), and poly(lactic-co-glycolic acid) (PLGA), providing excellent biocompatibility in living organisms 20. PLGA, which can be broken down into water and carbon dioxide, is highly biocompatible. The preparation of PLGA microspheres of varying sizes and morphologies can be achieved through techniques such as microemulsion, spray drying, and microfluidics 21. These microspheres are crucial for facilitating cell adhesion and drug delivery.

Traditional PLGA microspheres are categorized into solid and porous types 22. Owing to their dense surface and internal structure, solid microspheres limit their interaction with cells and biological tissues. This results in insufficient adhesion sites for cell attachment and a lack of internal space for cell proliferation. In contrast, porous microspheres have greater porosity and rougher surface topography. Furthermore, compared with other advanced functional microsphere designs (e.g., polymeric self-assemblies, probiotic-loaded or growth factor-eluting systems) 23-25 (Table 1), porous microspheres significantly enhance cell-material affinity, promoting the adhesion, growth, proliferation, and inward migration of cells. However, traditional porous microspheres exhibit limitations such as single porosity and limited drug loading capacity 26. This restricts their ability to be designed and controlled with respect to size and internal fine structure. Consequently, these porous microspheres possess limited pores and lack seamless connections between the surface and internal pore structure. Therefore, it is necessary to create a method that enables accurate design and control of both the surface and internal structures of microspheres. This technique would enable the production of porous microspheres with highly open pore structures, thereby advancing their effectiveness and potential applications 27. Furthermore, the application of previous PLGA microspheres in drug delivery has been significantly limited because of their low loading and release efficiency of hydrophobic drugs.

**Scheme 1:**
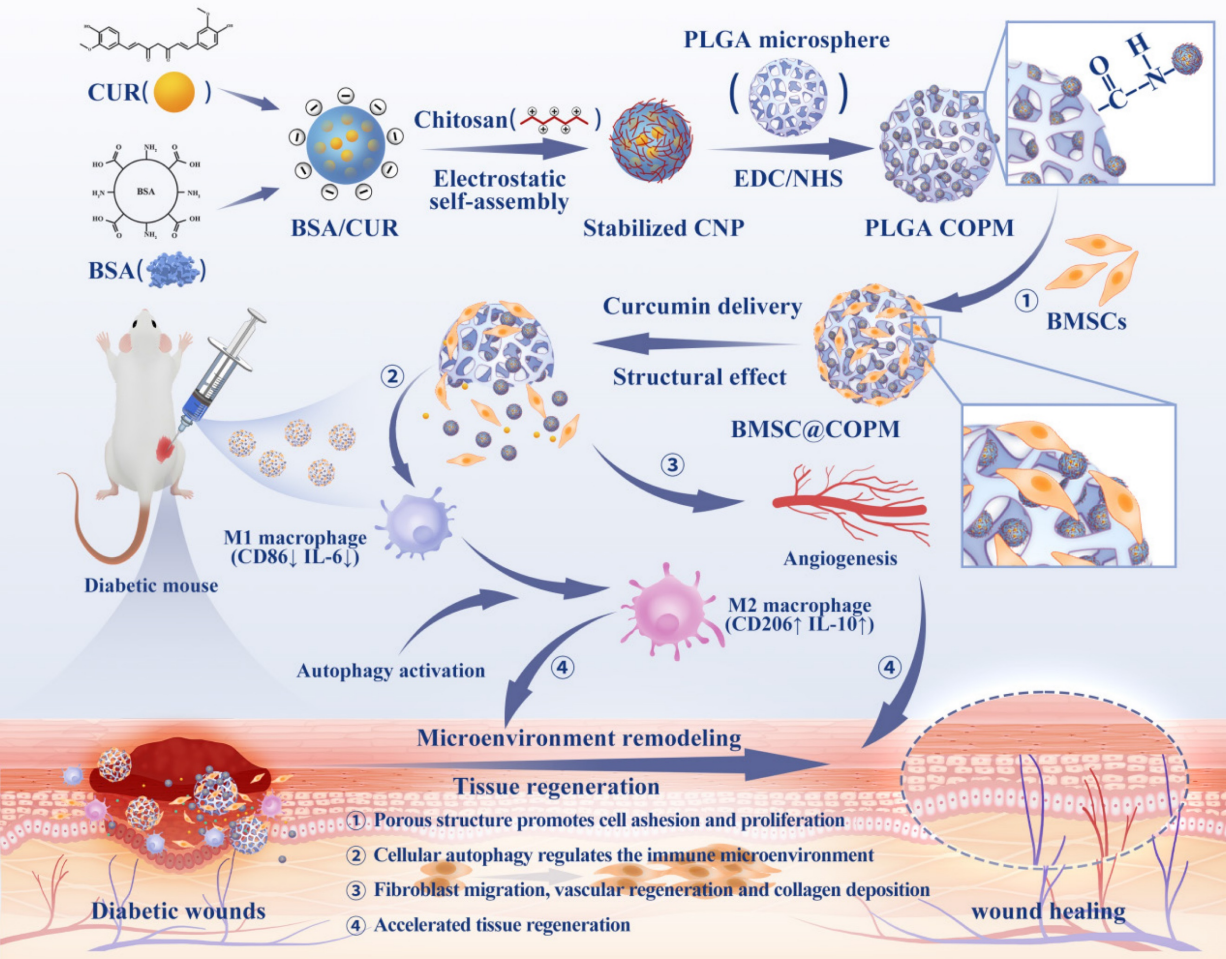
Microspheres with open pores made from poly (lactic-co-glycolic acid) (PLGA) were created to hold curcumin and BMSC for healing wounds in diabetic mice. Curcumin was complexed with bovine serum albumin (BSA) and stabilized with chitosan to form nanoparticles, which were then incorporated into the PLGA microspheres. These microspheres release curcumin, targeting inflammation resolution and modulating the wound microenvironment through cell autophagy. The porous architecture facilitated stem cell adhesion and proliferation, thereby promoting cell differentiation. The synergistic action of the microspheres, curcumin, and stem cells was observed to enhance wound vascularization and collagen synthesis.

In this study, we developed multifunctional absorbable PLGA microspheres to support stem cells and curcumin (BMSC@COPM). A combination of gas-assisted volatilization, microemulsion, and surface alkali treatment was employed. The hydrophobic agent curcumin is encapsulated with bovine serum albumin to introduce a dual-release mechanism involving both diffusion and degradation, thereby enhancing hydrophobic drug delivery. This innovative approach is more effective than previous approaches, as it allows the modulation of the activity of curcumin and prolongs its release duration. Moreover, the granular topography of our system enhanced cell adhesion and proliferation. This innovation has proven effective in reducing inflammation at the wound location and enhancing wound healing (Scheme 1). BMSC@COPM stimulates angiogenesis by regulating hypoxia-inducible factor 1α (HIF-1α) expression, which supports the formation of new blood vessels in the wound area. Recently, it has been reported that autophagy plays an essential role in various phases of refractory wound healing 28. Specifically, in the inflammatory phase, autophagy has an anti-infection effect and negatively regulates the inflammatory response. In the proliferative phase, autophagy plays a role in preventing apoptosis and oxidative stress and promotes cell survival 28. The process of autophagy in vascular endothelial cells aids in wound angiogenesis, facilitating the re-epithelialization of wounds. In the remodeling phase, the development of hypertrophic scars is affected by the autophagy of fibroblasts 29. Interestingly, BMSC@COPM induced autophagy in cells, leading to their polarization into the M2 phenotype, effectively modulating the wound microenvironment. Moreover, BMSC@COPM was essential in managing collagen network regeneration and aiding wound healing. The significant outcomes highlight the promising biomedical applications of BMSC@COPM in wound healing.

## Materials and Methods

### Materials

PLGA was obtained from Zhejiang Bangyao Biomaterials Co., Ltd. (Zhejiang, China). Dichloromethane (DCM) and anhydrous alcohol were got from Shanghai Lingfeng Chemical Reagent Co., Ltd. (Shanghai, China). Poly (vinyl alcohol) (PVA), ammonium bicarbonate (NH_4_HCO_3_), sodium hydroxide (NaOH), chitosan, bovine serum albumin, glutaraldehyde and acetic acid were purchased from Aladdin Bio-Chem Technology Co., Ltd. (Shanghai, China). Curcumin and Streptozotocin were purchased from Beijing Solarbio Science (Beijing, China). The MesenCult™ expansion kit (Mouse) was purchased from Stemcell Technologies (Vancouver, Canada). The Calcein/PI Cell Viability/Cytotoxicity Assay Kit was purchased from Beyotime Biotechnology Co., Ltd. (Shanghai, China). The RNA extraction kit was purchased from Omega Bio-Tek, Inc. (Doraville GA, USA). A high-capacity cDNA reverse transcription kit and SYBR Green Master Mix were purchased from Roche Pharmaceutical Ltd. (Basel, Switzerland). The antibody against LC3A/B was obtained from Cell Signaling Technology (Boston, MA, USA). P62 was bought from Abcam (Cambridge, UK). Antibodies against -HIF-1α, -α-SMA, -VEGF, -CD206, -CD86, CD31 were purchased from Cell Signaling Technology (Boston, MA, USA). p-AKT, p-PI3K and p-mTOR antibodies (Affinity, Hong Kong, China) were used. VEGF, IL-10, TGF-β and IL-6 ELISA kits and EDTA (0.5 M) were booked from Beyotime Biotechnology Co., Ltd. (Shanghai, China). Chloroquine and rapamycin were brought from MedChemExpress LLC (Shanghai, China). The mouse embryonic fibroblast line NIH-3T3 and the macrophage-like cell line RAW 264.7 were donated by the medical research center of Mudanjiang Medical University. We ordered human umbilical vein endothelial cells (HUVECs) from Shanghai Diyiming Biotechnology Co., Ltd. (Shanghai, China).

### Preparation of PLGA porous microspheres

PLGA porous microspheres were synthesized via microemulsion and surface treatment techniques. Initially, PLGA was dissolved in DCM and combined with an aqueous solution of ammonium bicarbonate (NH_4_HCO_3_). The primary emulsion was generated through high-speed mixing at 800 rpm for 10 min under room temperature condition. This primary emulsion was subsequently gradually transfer to the aqueous phase, then they were stirred at 800 rpm for 1 h to ensure complete emulsification. After an overnight stirring at room temperature, ammonium bicarbonate of the mixture decomposed completely, releasing NH_3_ and CO_2_ and forming interconnected pore structures within and on the surface of the microspheres. The resulting microspheres were designated porous PLGA microspheres (PMs). The solid microspheres without NH_4_HCO_3_ were named MS. A mixture of 0.25 M NaOH solution and ethanol (25 mL) was prepared to increase the surface and internal pore sizes. The PLGA PM from the previous step was immersed in this alkaline solution for a specific duration. Following washing with deionized water more than three times, the microspheres were freeze-dried and designated as PLGA open porous microspheres (OPMs) for use in subsequent experiments.

### Synthesis of curcumin-coated nanoparticles

Curcumin-coated nanoparticles (CNPs) were prepared via the desolvation method. Bovine serum albumin (BSA) and curcumin (CUR) were dissolved in ultrapure water and anhydrous ethanol, respectively. The curcumin solution was then added dropwise into the BSA solution while stirring. After 2 h of stirring at room temperature, glutaraldehyde was added to induce crosslinking, and stirring was continued. This led to the formation of negatively charged protein nanoparticles coated with curcumin after 4 h of stirring. To ensure the stability of the nanoparticle structure, chitosan with a positive charge was introduced by adding an acetic acid solution of chitosan into the mixture and stirring overnight. Stable nanoparticles were formed by electrostatic attraction between negatively charged and positively charged chitosan. The crude product of CNPs was obtained by centrifugation at 12,000 rpm for 20 min, followed by washing with ultrapure water three times. The product was then freeze-dried and prepared for future.

### Preparation and characterization of the PLGA COPM

The grafting methods described in previous literature are referred to for grafting CNPs onto PLGA OPM. Briefly, 1-(3-dimethylaminopropyl)-3-ethylcarbodiimide (EDC, 40 mg) and of PLGA OPM (30 mg) were combined with 60 mg of NHS in 10 mL of MES buffer. The mixture was stirred at 37 °C under an argon atmosphere for 45 - 60 min, and the carboxyl group on the OPM was activated to form a stable active ester intermediate. After activation, OPM and CNPs were dispersed in ultrapure water and stirred at 37°C under an argon atmosphere overnight. The mixture was washed with ultrapure water for three times and freeze-dried for further use. This product was named PLGA COPM.

The surface morphology and size of various microspheres were observed under a transmission electron microscopy (JEM-2100, Japan) and a scanning electron microscopy (SEM, Hitachi, S-3400 N, Japan). The sample's hydration size and zeta potential were measured using a Malvern laser particle size analyzer (Nano-ZS, Malvern). The water contact angles of the different microsphere samples were determined via a contact angle meter (JC2000D2, China). Fourier transform infrared spectroscopy was used to analyze the functional groups and chemical structures of the raw materials and microsphere samples. The polymer structure was conducted to assess changes in during microsphere preparation via differential scanning calorimetry (DSC) analysis (DSC2910, USA). The glass transition temperatures of MS, PLGA PM, and PLGA OPM were measured. Additionally, the water absorption properties of the microspheres were compared via the dry‒wet weight difference method in deionized water.

### *In vitro* degradability of microspheres

To assess the degradation performance of different microspheres, 20.0 mg of the microspheres PLGA/MS/PM/OPM were precisely weighed and immersed in 10.0 mL of PBS. During the degradation experiments, the samples were kept on a constant-temperature shaking table at 37 °C and 80 rpm. The microspheres were removed from the degradation solution on days 1, 4, 7, 10, 14, 21, 28, 35, and 42 of the degradation process. At each time point, there were 3 parallel samples for each microsphere type. A pH meter accurately measured the pH and changes in the degraded solution after separation. The separated microspheres were thoroughly cleaned with ultrapure water to eliminate attached inorganic salt particles. After freeze-drying, the samples underwent molecular weight analysis using gel permeation chromatography (GPC). Additionally, the degradation process morphology of the microspheres was observed via SEM.

### *In vitro* drug loading and release of microspheres

10.0 mg of PLGA COPM microspheres were dissolved in DCM. and then the absorbance of the solution was recorded. The mass m_0_ of the drug in the 10.0 mg microsphere was calculated via the following equation to determine the encapsulation rate (EE) and drug loading rate (DL) of the microsphere:


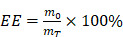

(1)


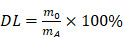

(2)

where *m_0_* represents the mass of the drug in the drug-harboring microsphere sample, *m_T_* represents the initial mass of the drug, and *m_A_* represents the total mass of the drug-harboring microsphere.

A quantity of 10.0 mg of drug-loaded PLGA COPM microspheres was mixed with 2.0 mL of PBS, put into a 10,000 kDa dialysis bag, and then placed in 8.0 mL of PBS (at least 3 parallel samples were examined). The samples were protected from light and placed in a constant-temperature oscillating chamber at 37 °C and 80 rpm for the release experiments. 300 μL of release solution was taken and transferred to 96-well plates (100 per well) at different time points (0.5, 1, 2, 3, 4, 5, 7, 8, 10, 12, 14, 16, 18, 20, 24, 28, 32, and 36 d). Meanwhile, 300 μL of PBS was added again to ensure that the total amount of released solution was always 10.0 mL. The absorbance of the solution in the orifice plate was measured, and the drug release rate was calculated via an enzyme-labeled instrument at the detection wavelength.

### *In vitro* cytocompatibility

After a 24 h sterilization process via ultraviolet irradiation, the three types of microspheres were dispersed in Dulbecco's Modified Eagle's medium (DMEM), which contain 10% FBS and 1% penicillin‒streptomycin. Then they were prepared as a microsphere suspension (100 μg/mL). Concurrently, the NIH-3T3 cell suspension was diluted to a concentration of 3000 cells/mL. *In vitro* cytocompatibility test was executed via a microplate reader according to previously reported methods 30,31. The cell viability was calculated as follows:

Cell viability (%) =100% × 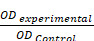
(3)

where *OD_experimental_* and *OD_control_* are the optical density values for the experimental groups and control groups, respectively.

Hemocompatibility was assessed as described in the literature 32. Specifically, erythrocytes of mouse blood were isolate via a centrifugal machine at 1000 rpm for 10 min. They were washed with saline and resuspended to achieve 5% (v/v). A 48-well plate was placed with the microspheres, and each well was added 1 mL erythrocyte suspension. After incubation and centrifuge, supernatant (100 μL) was transferred to 96-well plates and measured via a spectrophotometer at 450 nm. Meanwhile, we select 0.1% Triton X-100 as positive control and PBS as negative control. The percentage of hemolysis was calculated via the following formula:

Hemolysis (%) =100% × 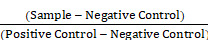


### Isolation of bone marrow mesenchymal stem cells

Newborn ICR mice purchased from Liaoning Changsheng Biotechnology Co., Ltd. (Benxi, Liaoning, China) (SCXK (Liao)2022-006) were euthanized with CO_2_ and soaked with 75% alcohol for 10 min. Their tibias and femurs were separated under sterile condition and then placed in preprepared anticoagulant buffer. After sampling, the anticoagulant buffer was removed, and type I collagenase solution was added. The BMSCs were isolated as previously reported methods 19. One day later, the adherent BMSCs were observed under a microscope for morphology and growth. Third- or fourth-generation cells were prepared in standby used.

### Cultivation of bone marrow mesenchymal stem cells

The culture medium in the T5 culture bottle containing the BMSCs was discarded. 3 mL of pancreatic enzyme digestion solution (0.25%) was added after the samples were washed with PBS. Then, the cells were digested in the incubator for 1 min. Subsequently, the digestion was stop by 3 mL of complete medium. The cell density was modified to 1×10^6^ cells/mL after centrifugation.

### Methods of cell culture

Human umbilical vein endothelial cells (HUVECs), mouse embryonic fibroblasts cells (NIH-3T3 and mouse mononuclear macrophages (RAW 264.7) were cultured as previously reported methods 19. HUVEC and NIH-3T3 were cultured with a 35 mM glucose environment to simulate the diabetic condition. When the cell density reached 80%, they were detached via trypsin and suspended in fresh cell medium for standby application.

### Microsphere pretreatment

A total of 5 mg of the microspheres was exposed to ultraviolet light and immersed in 75% ethanol for more than 12 h respectively. After a brief incubation period, the gelatin at the bottom of the 12-well cell culture plate solidified. The microspheres were transferred to the culture dish for next step use.

### BMSCs were cocultured with microspheres

The cells were inoculated on the open porous microspheres described above (each well contain about 100 μL of 2×10^6^ cells/mL cell suspension). The cells were cultured for 2h in order to ensure adhesion to the open porous microspheres. Afterward, 800 μL of complete medium was added and was refreshed every two days.

### Cell viability assay

Viability of the cells were evaluated via MTT and CCK-8 assays. Briefly, 4000 cells/mL were exposed to different substances (BMSC@MS, BMSC@PM, BMSC@OPM and BMSC@COPM) in 96-well plates (Costar, USA). Meanwhile, 35 mM d-glucose was used as the control. The remaining steps are as reported earlier 19. To establish the relative number of cells, the BMSCs were cultured (1.25 × 10^3^, 2.5 × 10^3^, 5 × 10^3^, 1 × 10^4^, and 2 × 10^4^) in 96-well plates, and the OD value was detected according to our previous reports 19. The different numbers of cells were evaluated according to the calibration curve.

### Bioluminescence assay

Bioluminescence assay was performed to assess the BMSCs survival in *in vivo*. BMSCs were transfected with Luc-GFP+ and were prepared BMSC@COPM and bioluminescence photons were measured according to our previous reports 19. Images were recorded on day 1, 2, 3, 4, 5 and 7 via small animal live imaging instrument (Tanon ABL-X6, Shanghai, China) was used, and photons of bioluminescence were measured in the area of interest.

### Scratch Test

To evaluate fibroblast migration in coculture with BMSCs with or without laden microspheres, a wound healing assay was carried out. NIH-3T3 cells or HUVECs (5 × 10^5^ cells/well) were seeded in 6-well plates with normal culture medium or culture medium containing 35 mM glucose for 24 h. A scratch was formed according to our previous reports 19. The alterations in scratch width (ΔScratch) were determined using this formula:

Wound area ratio (%) =100% × 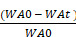


where *W_A0_* and *W_At_* refer to the starting wound area and the wound area at time *t*, respectively.

### Cell migration assay

To assess cell migration capability, a transwell migration experiment was performed. NIH-3T3 cells (2×10^4^ cells/mL) in serum-free DMEM were transferred to the chamber for a 6-hour. The upper and lower chambers were incubated separately with 300 μL and 700 μL of normal culture medium or culture medium containing 35 mM glucose for 36 h. After 36 h of incubation, the upper chamber medium was removed, and 300 μL of the co-culture product of each group of microspheres and BMSCs was added. After 24 h of incubation, the upper chamber membrane was fixed with 4% paraformaldehyde for 20 mins after two PBS washes. Then, it was stained with crystal violet dye for 15 mins. Cell migration was observed via a light microscope (Nikon, FHEIPSE Ti, Japan).

### Angiogenesis assay

In order to evaluate the angiogenesis ability of cells after treatment with different material.

HUVECs were used for the tube formation assay with or without different material. To simulate the diabetes model, the culture medium we used contained 35 mM glucose. Other steps were carried out according to our previous reports 19.

### Live/dead cell staining

Live/dead cell staining was executed when cell was seeded in microspheres for 3 and 5 days. A working solution for live/dead staining was prepared using 1 μL each of calcein AM (1000×) and PI (1000×), along with calcein AM/PI detection solution (700 μL). Then, the 24-well plate (300 μL per well) was incubated at 37°C for 45 min under light-protected. They were then observed under a fluorescence microscope, after being fixed in a 4% paraformaldehyde solution and washed three times with PBS. The growth of BMSCs on the four types of microspheres were observed via confocal fluorescence microscopy at the excitation wavelengths of 488 nm and 561 nm.

### Cell seeding in transwells

To establish a coculture model, transwells were used (Costar Corning, Kennebunk, ME, USA). BMSC cells and RAW264.7 cells were placed in the upper and the lower chamber, respectively. The whole procedure was performed in an aseptic environment to avoid contamination during the cell culture procedures. After coculturing the cells for about 24 hours, the RAW264.7 cells were extracted for immunofluorescence analysis.

### Real-time quantitative PCR

Inflammation-related mRNAs (TGF-β, IL-6, IL-10 and CD206) in RAW 264.7 macrophages were quantified via qRT‒PCR analysis. Additionally, the mRNAs expression which are angiogenesis-related were assessed in HUVECs (PDGF, FGF, and VEGF). Real-time PCR (qPCR) was conducted according to previous studies 19. Relative gene expression was calculated via the 2^-ΔΔ^CT method. The sequences of the primers used in the experiment are listed in Table 2.

### Western blotting

The protein expression of HIF-1α, VEGF, CD206 and CD86 were determined by Western blotting (WB) with GAPDH as control, according to the manufacturer's instructions. HIF-1α, VEGF, CD206, CD86, P62, LC3I/II, pAKT and pPI3K (1:1000) antibodies were used. Other steps were carried out according to our previous reports 19.

### Mouse model of diabetic wounds

Ninety male ICR mice were purchased from Liaoning Changsheng Biotechnology Co., Ltd. (Benxi, Liaoning, China) (SCXK (Liao)2024--003) and divided into 5 groups: the control group (n = 18), BMSC@MS (n = 18), BMSC@PM (n = 18), BMSC@OPM (n = 18), and BMSC@COPM (n = 18). Each group consisted of nine animals (6 at each time point), and the sample size was determined through scientific consideration and aligned with standard industry practices 19,33,34. This sample size was sufficient to ensure statistically meaningful results while minimizing unnecessary animal use, in accordance with the guidelines of ARRIVE 2.0. The study was approved by the Institutional Animal Care and Use Committee of Mudanjiang Medical University (ID number IACUC-20240815-202). All surgeries were performed under anesthesia via the carbon dioxide method. Animals were allocated to the treatment or control group through a computer-generated random sequence (GraphPad Prism 9.0) with stratification by body weight, which was performed independently by a researcher not involved in the experiment. The allocation scheme was sealed in opaque envelopes and remained unopened until the intervention began. All groups of mice were maintained on a regular diet. After fasting for 15 h, an intraperitoneal injection of sodium citrate buffer chain urea with streptozotocin (STZ,120 mg/kg) solution. Then, the fasting blood glucose levels of the ICR mice were measured for three consecutive days. If the fasting blood sugar level exceeded 16.7 mM during this period, insulin/glucose tolerance tests were assessed as previously reported 35,36. The results revealed that glucose tolerance and insulin tolerance of model were significantly worse than normal group. These findings indicated the type 1 diabetes mellitus mouse model were successful. A diabetic wound model was subsequently created by anesthetizing the mice with an 8 mm sterile hole punch, followed by disinfection before drug treatment.

### BMSC@COPM treatment *in vivo*

The mice were housed with a 12 h light/dark cycle rhythm at 22 °C. Each group received approximately 5 mg of the microsphere complex, which was administered subcutaneously in a total volume of 200 μL every other day four times according to a previously reported method 19. Each mouse was breed in one cage, individually. Changes in the wounds and health behavior of the mice was observed. Meanwhile, taking photos to assess development of wound healing. Venous blood was collected after anesthesia on days 7, 14, and 21, and the animals were euthanized by cervical dislocation after the administration of anesthesia with carbon dioxide. A portion of the tissues was fixed in a 4% formaldehyde solution, and the remaining tissues were preserved at -80 °C for subsequent use.

### Compatibility* in vivo*

A small piece of the main organ tissues (heart, liver, spleen, lung, kidney, test) were fixed in a 4% formaldehyde solution for 48h, then they were embedded in paraffin. Then, the hematoxylin and eosin (H&E) staining was carried out for pathomorphological assessment. Meanwhile, the blood samples (obtained as the method described above) were subsequently analyzed via an automated biochemical analyzer (Beckman Coulter, Brea, CA, USA). Biochemical indicators measured were: urea nitrogen (UREA), creatinine (CREA), CO_2_ combining power (CO_2_CP), uric acid (UA), alanine aminotransferase (ALT), aspartate aminotransferase (AST) and total bilirubin (TBIL) 36.

### Histological staining

We employed established staining methods such as H&E, Masson's trichrome, and Sirius Red on tissue samples to evaluate histopathological changes during skin regeneration according to our report 36. Pathological changes were assessed via H&E staining, while collagen deposition was examined via Sirius Red staining and Masson's trichrome.

The histopathological scoring methods about re-epithelialization, collagen deposition, mature granulation tissue and vascularization were assessed according to previous methods 37,38, which were divided four points: 0, 1, 2, 3 and 4. Zero (0) points were used for no (re-epithelialization, collagen deposition, mature granulation tissue and vascularization) tissue structure, and 4 points were used for varying degrees of appearance. Scores ranged from 0 to 4, with 0 indicating the most severe pathology and 4 representing excellent wound healing.

### Immunofluorescence (IF) and immunohistochemical (IHC) assay

The protein expression of CD31, HIF-1α, α-SMA CD206, CD86 and VEGF were conducted via IF and IHC assay as established protocols outlined in a previous study 39. The antibodies targeting α-SMA, CD31, HIF-1α, VEGF, CD86, and CD206 (at a dilution of 1:50) were used. Visual documentation was obtained by an optical microscope (Nikon FHEIPSE Ti; Japan) as well as a confocal microscope was used for fluorescent signal (Olympus FV3000; Tokyo, Japan).

### Enzyme-linked immunosorbent assay

An enzyme-linked immunosorbent assay (ELISA) was performed to evaluate the protein expression of IL-6, IL-10, VEGF and TGF-β in mice blood according to the manufacturer's instructions. Cell supernatants were obtained from RAW 264.7 cells, which were treated as described above. The detailed process follows the method previously reported 19.

### Autophagosomes were observed via transmission electron microscopy (TEM)

After being discarding the culture medium, the different groups of NIH-3T3 cells (Control, High glucose, BMSC@OPM and BMSC@COPM) were swiftly treated with 2.5% glutaraldehyde. The different cells were carefully scraped and were collected. The cells were treated with glutaraldehyde and were centrifugated at 1500 rpm for 3 min. Subsequently, the cells underwent a series of operations: were fixed in 2% OSO4, dehydrated, embedded, being ultrathin sliced. Then, the ultrathin slices were stained with uranium acetate and lead citrate. Finally, they were detected under a transmission electron microscopy (JEM-1230, Tokyo, Japan) to search autophagosomes.

### Statistical analysis

The study employed Graphpad Prism 9.0 (San Diego, CA, USA) for the data analysis, which are presented as the means ± SDs. One-way analysis of variance (ANOVA) with Tukey's multiple comparison test or unpaired student's t test was used. Statistical significance was considered as *P* < 0.05.

## Results and discussion

### Characterization of PLGA microspheres

**Figure 1:**
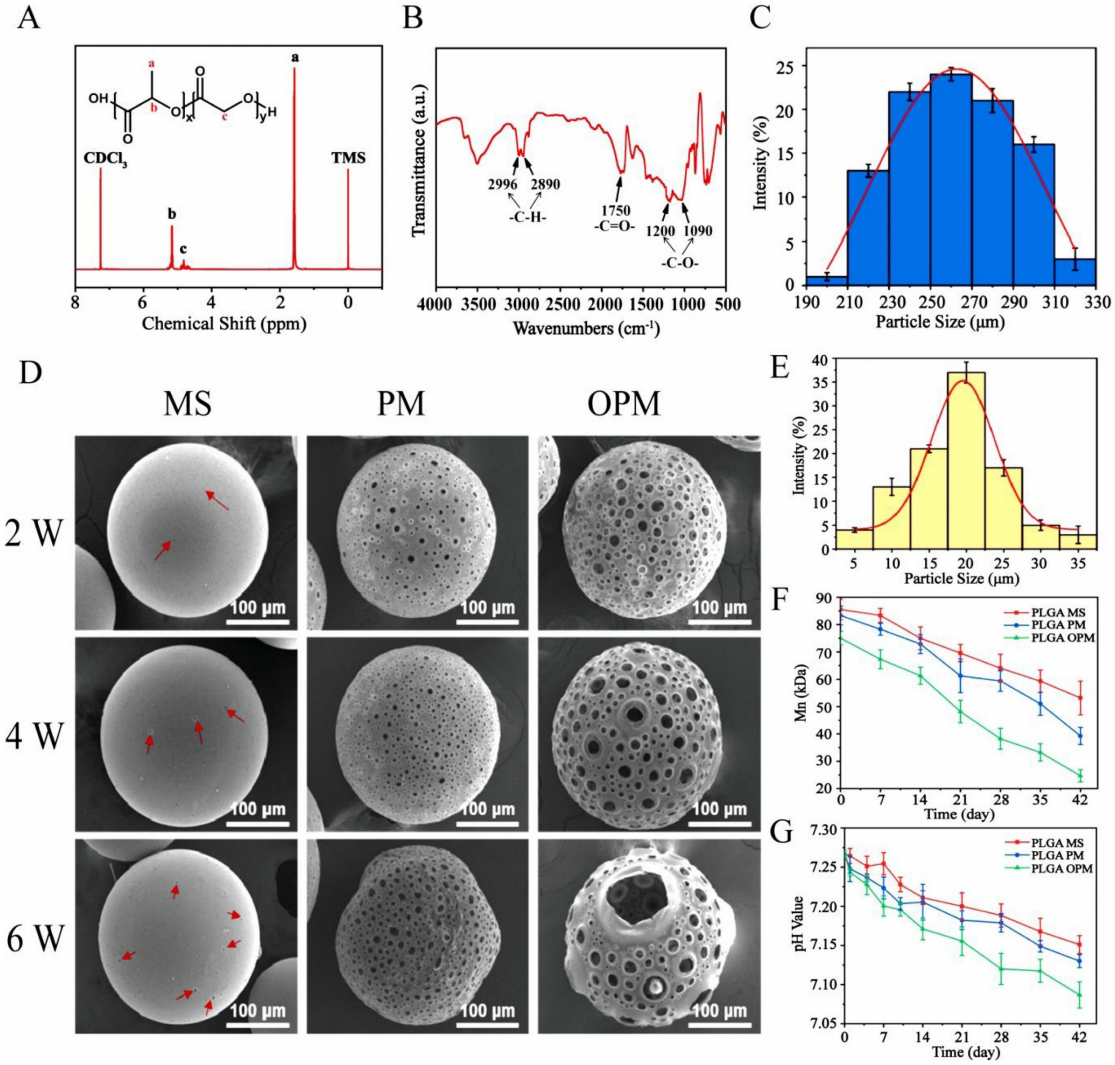
Chemical structure and surface topography analysis of the microspheres. (A) 1H NMR. (B) FTIR spectrum. (C) Size distribution of the microspheres. (D) Changes in surface morphology during the degradation of microspheres detected by SEM. (E) Pore size distribution of the microspheres. Changes in the (F) molecular weight and (G) pH during the degradation of the microspheres. All data shown in the figure are presented as the means ± SDs (n = 3).

The ^1^H NMR spectrum of PLGA exhibited a characteristic chemical shift for the methyl group (9H, m, CH_3_) on the polymer chain at δ = 1.56 ppm, whereas the submethyl group (1H, m, CH) was observed at δ = 5.19 ppm 40. Additionally, the methylene group (2H, m, CH_2_) on the PLGA backbone exhibited a chemical shift at δ = 4.82 ppm (Fig. 1A). In the FTIR spectrum of PLGA, absorption peaks appeared at 2996 and 2890 cm^-1^ 38, corresponding to the stretching vibrations of C-H (Fig. 1B). The stretching vibration of C-O was indicated by the peaks at 1200 and 1090 cm-1, while the peak at 1750 cm^-1^ verified the presence of a C = O group. Moreover, SEM analysis showed that the microspheres had sizes predominantly between 230 and 290 µm, suggesting a high level of uniformity (Fig. 1C). Through the optimization of various conditions, PLGA microspheres with a uniform porous structure were obtained (Fig. S1). In the absence of NH_4_HCO_3_, the PLGA microspheres exhibited a nonporous and smooth surface (MS). However, the incorporation of NH_4_HCO_3_ at a concentration of 10% (w/w) resulted in a surface with numerous evenly distributed pores (PMs) (Fig. 1D). Alkali treatment of the microspheres increased both the surface and internal pore sizes, thereby increasing their cellular affinity. Notably, a treatment time of 7 min yielded PLGA OPM microspheres with an interconnected porous structure. The porous surfaces of these microspheres are composed of large pores with diameters ranging from 15 to 25 µm (Fig. 1E) and micropores less than 10 µm in size. The larger pores facilitated cell migration into the interior, whereas the smaller micropores allowed oxygen and nutrient transport (Fig. S2). For cell loading and injectable uses, microspheres should have a size range of 200 to 300 µm. The average sizes of particles produced by magnetic stirring at 600, 800, and 1000 rpm were 326.58 ± 42.31 µm, 223.76 µm. Therefore, a magnetic stirring speed of 800 rpm was selected (Fig. S3A-C). Additionally, we analyzed the effect of the stirring rate on the morphology of the microspheres and controlled the reaction temperature and gas type. Seven different concentrations of the solutions were labeled with enzymes, and the linear fitting equation for CUR was calculated. The encapsulation efficiency (EE) of curcumin by the microspheres was determined to be 79.57%, and the drug loading (DL) efficiency was calculated to be 7.62% (Fig. S3D-E).

After a two-week degradation period, the MS microspheres maintained a relatively smooth surface with minimal changes. In contrast, both the PM and OPM microspheres exhibited increased surface porosity. As degradation progressed to 4 and 6 weeks, the MS microspheres began to show small holes, as indicated by the red arrows. Over time, the number of holes on the MS microspheres gradually increased. Similarly, the PM and OPM microspheres experienced an increase in the number of surface holes and degradation of the interhole connection structure. Notably, by the sixth week, the PM and OPM microspheres tended to collapse due to the degradation of the interpore structure. Consequently, the microspheres shrink in size, and the interior of the OPM microspheres completely degrade into a hollow state.

The degradation process of the microspheres over a 6-week period is illustrated in Fig. 1F and 1G. The pH of the supernatant consistently remained above 7, indicating that the acidic byproducts of the degradation process did not disrupt the body's equilibrium environment. A comparison of the degradation rates of the three types of microspheres revealed significant differences. The degradation rate of the PLGA MS microspheres was the slowest, followed by that of the PLGA PM, with the PLGA OPM showing the fastest degradation. Over 6 weeks, the average molecular weight of the microspheres decreased by 32, 44, and 51 kDa, corresponding to reductions of 37.84%, 52.90%, and 67.09%, respectively (Fig. S4). These differences are attributed to the open, interconnected porous structure of the PLGA OPM, which allowed the degradation solution to penetrate the microspheres, enabling simultaneous interior and surface degradation, as opposed to the PLGA MS, which only initiated degradation from the surface.

**Figure 2:**
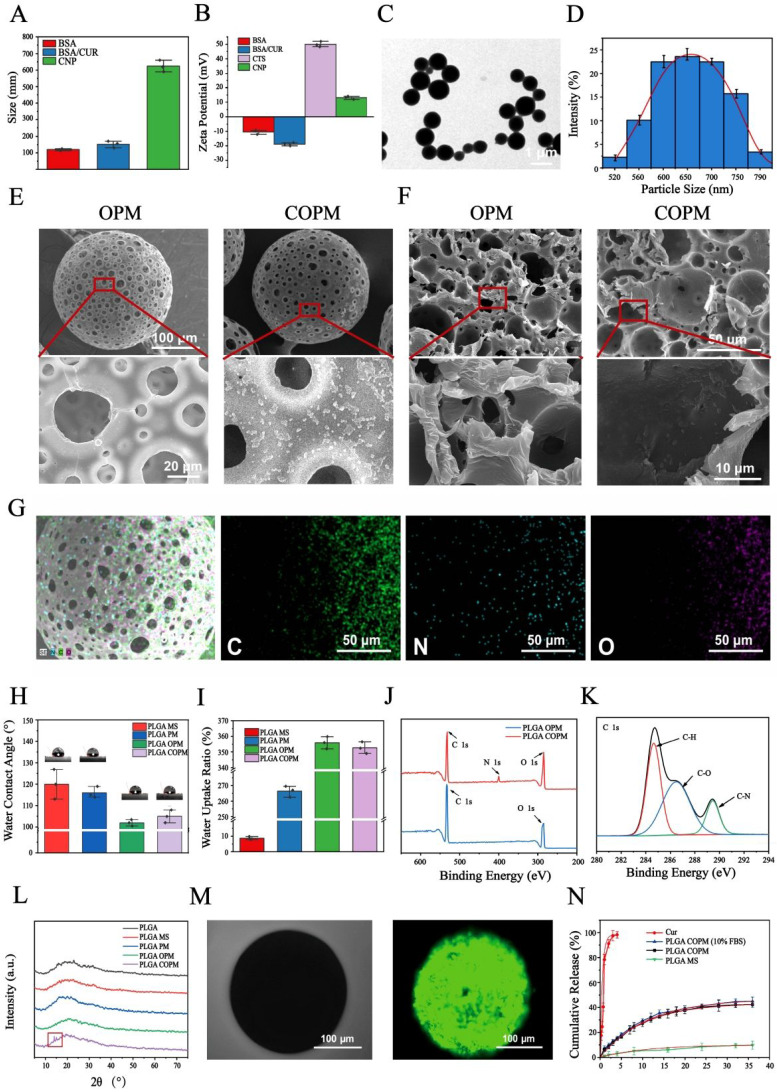
Characteristics of the CNPs and PLGA COPM. (A) Size, (B) zeta potential, (C) TEM micrograph and (D) size distribution of CNPs. (E) SEM surface micrograph and (F) internal micrograph of PLGA OPM and PLGA COPM. (G) EDS spectra of PLGA COPM. (H) Water contact angle and (I) water uptake ratio of the PLGA MS, PLGA PM, PLGA OPM and PLGA COPM. (J) XPS and (K) carbon peak (C 1 s) high-resolution spectra of the PLGA COPM. (L) XRD curves of different microspheres. (M) Fluorescence image of the curcumin distribution in the microspheres. (N) Tracking curcumin release cumulatively over different time periods. All data shown in the figure are presented as the means ± SDs (n = 3).

Nanoparticle surface charge and size significantly influence the loading and the release of drug. In this study, negatively charged BSA was coated with CUR to form nanoparticles with an average diameter of 159.43 ± 8.23 nm. In contrast, chitosan, which is positively charged, assembled into nanoparticles with a relatively large average particle size of 628.96 ± 18.49 nm (Fig. 2A). The polymer coating substantially increased the size of the nanoparticles and altered the surface charge from negative to positive, confirming the successful encapsulation of chitosan (Fig. 2B). Transmission electron microscopy (TEM) confirmed that the nanoparticles exhibited a smooth, spherical structure with good dimensional uniformity and dispersion. According to statistical analysis, the average size of the coated nanoparticles was found to be 643.51 ± 56.31 nm (Fig. 2C and 2D). The surface and internal morphology and structure of the porous microspheres before and after nanoparticle grafting were examined using scanning electron microscopy (SEM) (Fig. 2E). After grafting, spherical nanoparticles were observed on the smooth surface of the porous microsphere PLGA OPM, and a connected porous structure with a smooth inner wall was evident upon cross-sectioning the microspheres (Fig. 2F). The smooth inner surface of the grafted nanoparticles also featured numerous spherical nanoparticles. These observations confirmed the successful grafting of CNPs onto the microspheres and the synthesis of the PLGA COPM. Furthermore, energy dispersion spectroscopy (EDS) analysis demonstrated the even distribution of nitrogen on the surface of the PLGA COPM, indicating the successful presence and distribution of CNP nanoparticles on the microspheres (Fig. 2G). The water contact angles of the PLGA MSs and PLGA PMs were 120.51 ± 3.27° and 114.96 ± 3.40°, respectively. Moreover, that of the PLGA OPM was significantly lower at 102.98 ± 2.50°, indicating a noticeable improvement in the hydrophilicity of the PLGA OPM (Fig. 2H). The improved hydrophilicity can provide better cell affinity for the material, and the reduced contact angle also suggests the possibility of molecular chain breakage and the formation of hydrophilic carboxylic groups or hydroxyl groups during pore formation. The increase in water absorption by the microspheres was more pronounced than the decrease in contact angle. As shown in Fig. 2I, the water absorption of the PLGA MS was only 8.39 ± 1.27%, which was significantly lower than that of the PLGA PM (268.00 ± 2.99%), whereas the water absorption of the PLGA OPM reached as high as 356.97 ± 3.29%.

The grafting reaction was further validated by X-ray photoelectron spectroscopy (XPS) analysis, which showed a clear signal peak for the C‒N bond on the PLGA COPM surface. Both PLGA OPM and PLGA COPM presented peaks for carbon and oxygen, whereas only PLGA COPM presented nitrogen peaks (Fig. 2J and 2K). On the basis of these results, CNPs were successfully grafted onto the PLGA OPM, leading to the successful preparation of the PLGA COPM. X-ray diffraction (XRD) characterization was performed on PLGA raw materials and different microspheres to investigate the influence of the microsphere preparation process on the chemical and crystalline structure of the material, the grafting process of nanoparticles, and the introduction of drugs. Semicrystalline polymers such as PLGA typically exhibit a distinctive pattern resembling a steamed bun peak in the XRD pattern, as shown in Fig. 2L. Apart from those of the PLGA COPM, the other curves display the same steamed bun peak shape. In contrast, PLGA COPM additionally shows two diffraction peaks at 14.7° (11-1 crystal plane) and 15.3° (20-2 crystal plane), corresponding to the characteristic peaks of curcumin. This confirmed the successful introduction of curcumin into the microspheres through the grafting reaction.

PLGA OPM without drug loading did not exhibit any fluorescence in the FITC channel or the DAPI channel. As shown in Fig. 2M, only the spherical structure of the microspheres could be observed under bright field illumination. In contrast, the PLGA COPM displayed uniform green fluorescence distributed on the microspheres, indicating the successful loading of curcumin both inside and outside the microspheres. During the 35-day release cycle, compared with that of solid nonporous microspheres, the release of curcumin by the porous microspheres was slow and sustained, with 15% released on the 5th day and 45% released on the 35th day, indicating a controlled release profile, and this dose is the appropriate dose for curcumin in wound treatment 42. The longer-lasting release of curcumin also shows promising therapeutic potential for the treatment of refractory and recurrent wounds. In addition, curcumin maintained a similar release curve in 10% FBS-supplemented DMEM. This slow and sustained release phenomenon can be attributed to the double coating characteristics, wherein curcumin is first encapsulated in BSA nanoparticles and then grafted onto the microspheres, effectively controlling the activity and controlled release of curcumin (Fig. 2N). Curcumin combined with BSA is a well-established method for prolonging drug release. The factors influencing curcumin release include pH and the mixing ratio 43,44. In this study, we grafted nanoparticles formed by curcumin and BSA onto porous PLGA microspheres to achieve a multistage double-coating prolongation of drug release. Specifically, following tissue trauma, vascular damage impairs oxygen delivery, leading to a slightly acidic pH environment within the wounded tissue 45. The degradation of microspheres generates a similar slightly acidic microenvironment, which interacts with the anaerobic metabolism of the traumatized tissue's blood vessels. The cumulative release curve and release kinetics of curcumin demonstrated that this unique slightly acidic environment effectively promoted the slow release of curcumin. Additionally, the combination of curcumin and BSA leads to a sustainable release process that regulates the tissue microenvironment of the wound, modulates inflammation, and promotes wound healing. Conventional PLGA microspheres exhibit inefficiencies in loading hydrophobic drugs and have limited functional effects. For example, Shahbazi et al. optimized the loading of glatiramer acetate in PLGA microspheres to prolong its slow release. However, the repair efficacy and overall functionality of these microspheres remain suboptimal 46. The present study combined the hydrophobic drug curcumin with bovine serum albumin to address these limitations and grafted it onto porous PLGA microspheres. This approach not only increased the loading capacity for hydrophobic drugs but also achieved a 35-day sustained release of the drug. Furthermore, the microspheres remodeled the tissue trauma microenvironment, enhanced vascular and collagen regeneration, and effectively promoted trauma repair.

### Open porous microspheres have a high loading capacity and protective effects on BMSCs

**Figure 3:**
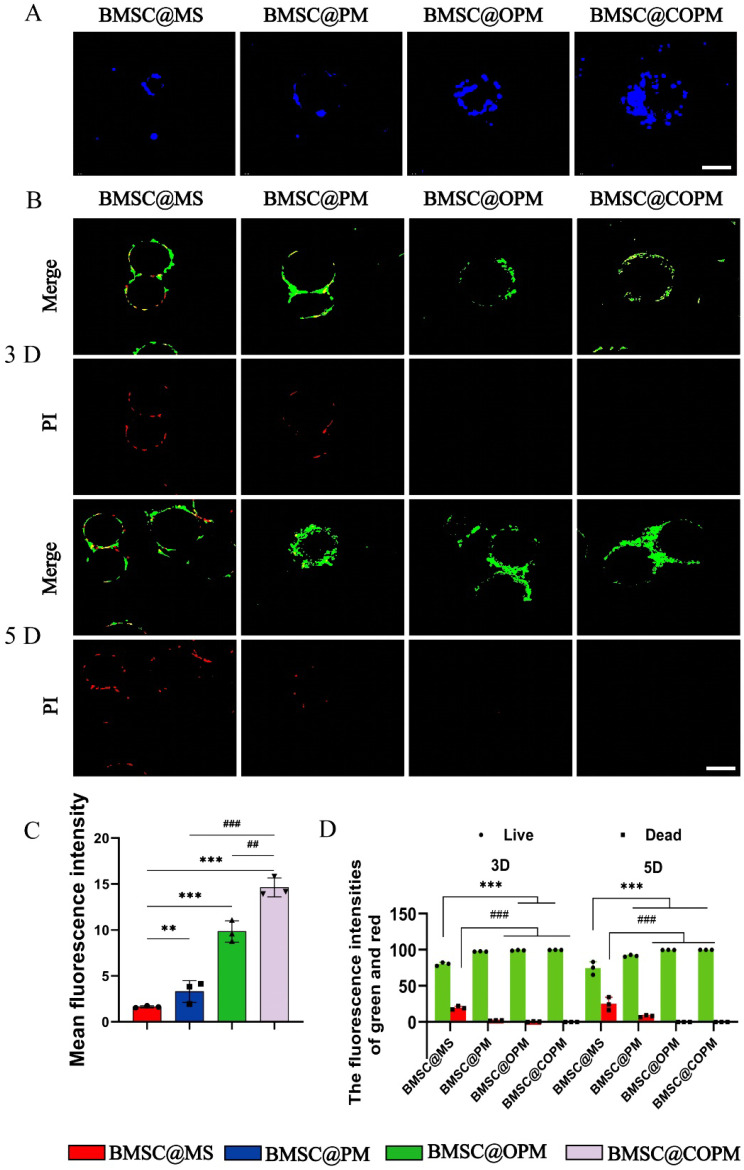
Adherence and survival of BMSCs on open porous microspheres. (A) After the microspheres were cocultured with BMSCs for 24 h, they were stained with DAPI (scale bars: 50 µm). (B) Changes in fluorescence intensity were observed in live cells (green) and dead cells (red) for each group on day 3 and day 5 via a confocal microscope (scale bars: 100 μm). (C) Measurement of the fluorescence intensity of (A) in quantitative terms. (D) Analysis of the fluorescence intensities in (B) was conducted quantitatively. Compared with the control group, ^**^P < 0.01 and ^***^P < 0.001; compared with the BMSC@COPM group, ^##^*P* < 0.01. All data shown in the figure are presented as the means ± SDs (n = 5).

The earliest research of advanced biomaterials and tissue regeneration engineering has greatly improved the therapeutic capabilities of BMSCs in healing skin wounds 47. To elucidate the impact of the microsphere architecture on BMSCs, we encapsulated BMSCs with various microsphere formulations (termed BMSC@MS, BMSC@PM, BMSC@OPM and BMSC@COPM) and observed their interactions. As Fig. 3A shown, the BMSCs predominantly adhered to the surface of the MS and PM microspheres, with notable internalization found in the OPM and COPM. The result indicates that an increase in the pore size of the microsphere surface is correlated with increased BMSC adhesion. Significantly, BMSC@COPM showed a much higher fluorescence intensity, indicating that the large internal space of the COPM microspheres increased BMSC survival, an effect further amplified by the inclusion of curcumin (Fig. 3C). We established the loading capacity of BMSC cells according to methods described in our previous report 19. As shown in Fig. S5, open porous microspheres have a high loading capacity and protective effects on BMSCs, and the loading capacity of COPM is 6.8
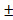
0.1 × 10^3^ per 5 mg/mL.

To determine the ability of the BMSC@COPM to prolong cell survival and proliferation, live/dead cell staining was performed on microsphere-encapsulated BMSCs on the 3rd and 5th days (Fig. 3B and 3D). The green fluorescence intensity increased over time in both the BMSC@OPM and the BMSC@COPM groups, with no detectable red fluorescence, indicating robust cell viability. These conclusions stress the importance of open porous structures in improving the survival and retention of BMSCs, which are vital for successful wound healing 48.

BMSC function can be compromised by excessive oxidative stress in the microenvironment of diabetic wounds 49. We assessed the protective effects of the four microspheres by exposing the BMSCs to a 500 μM H_2_O_2_ environment (Fig. S6A). Cell activity in all microsphere groups was significantly higher compared to the control group, with BMSC@COPM showing the greatest enhancement (Fig. S6B). These findings suggest that BMSC@COPM creates a protective niche that promotes BMSC survival.

### Biocompatibility of BMSCs and microspheres: *In vivo* and* in vitro assessment*

Biocompatibility is a paramount criterion for biomaterials in the clinical realm of wound dressing application. Utilizing established research methodologies, we conducted a comprehensive evaluation of the biocompatibility of the microspheres both *in vivo* and *in vitro* 19. The study results indicated that there were no significant differences among different groups, suggesting that the microspheres were highly compatible with cells and non-toxic (Fig. S7). The hemolysis test results showed that hemolysis rates for various concentrations of COPM or OPM were under 5%, indicating good blood compatibility (Fig. S8 A, B).

The toxicity of the BMSC@COPM *in vivo* was further examined by administering it to full-thickness wounds in mice. H&E staining showed that the major organs (heart, liver, spleen, lung, kidney, and testis) were undamaged in the groups treated with microspheres (Fig. S9A). The levels of liver and kidney function markers, including alanine aminotransferase (ALT), aspartate aminotransferase (AST), urea nitrogen (UREA), creatinine (CREA), carbon dioxide combining power (CO_2_CP), AST/ALT, total bilirubin (TBIL) and uric acid (UA), were assessed. The results were not statistically significant (Fig. S9B). The results suggested that the microspheres were non-toxic over extended therapeutic use, underscoring their promise as biomaterials for healing diabetic wounds.

### Enhanced fibroblast proliferation and migration by BMSC-laden microspheres

**Figure 4:**
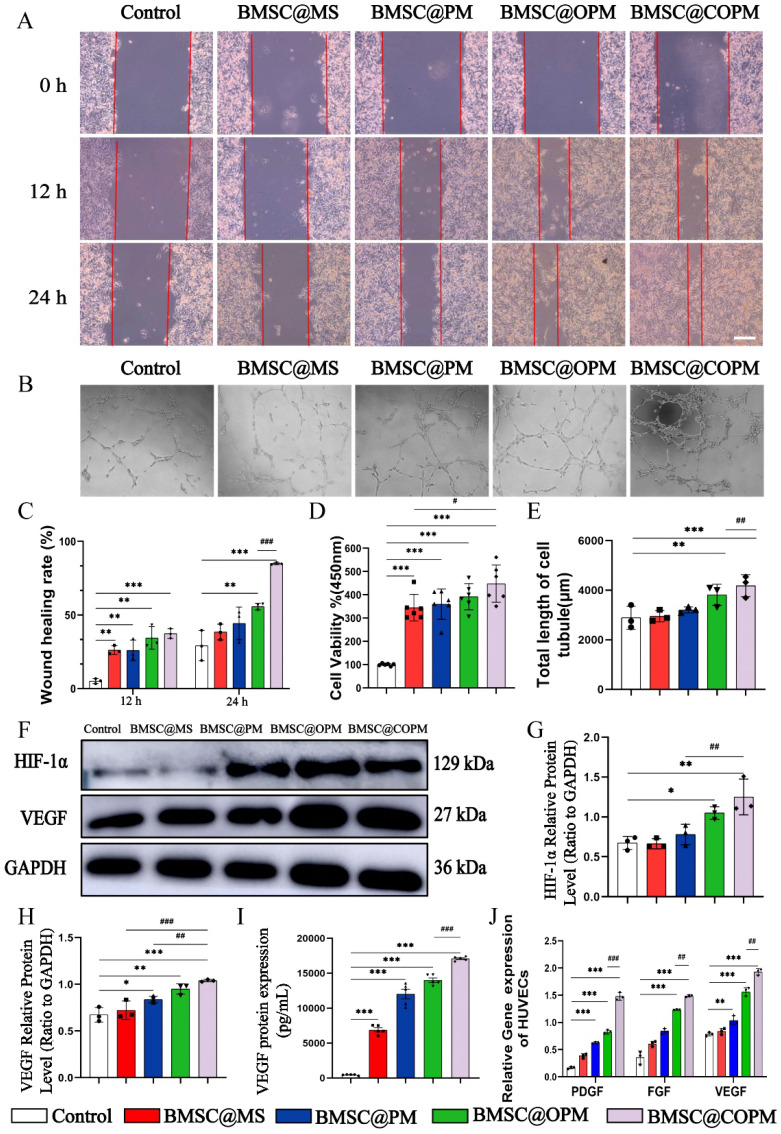
Angiogenesis and migration assays *In vitro*. (A) Images representing scratch test of NIH-3T3 cells in a high-glucose environment. (B) Images representing the *in vitro* tube formation test and measurement of the total length of HUVECs exposed to BMSC@COPM in a high-glucose setting (scale bars: 100 μm). (C) Quantitative analysis of the scratch test results. (D) Under high-sugar conditions, BMSC@COPM promoted the proliferation of HUVECs, and cell viability was determined by a CCK-8 assay (n = 6). (E) Quantification of total length of HUVECs. (F) The representative WB images of HUVECs post-treatment with microspheres loaded with BMSCs. (G and H) Quantitative evaluation of HIF-1α and VEGF. (I) The expression of VEGF protein in HUVECs measured via ELISA. (J) In HUVECs, the levels of angiogenesis-related mRNAs (PDGF, FGF, and VEGF) were quantified using qRT-PCR. *vs* the control group, ^*^P < 0.05, ^**^P < 0.01 and ^***^P < 0.001; *vs* the BMSC@COPM group, ^##^*P* < 0.01 and ^###^*P* < 0.001. The data shown in the figure are presented as the means ± SDs (n = 3, except D).

Under elevated glucose conditions, wound healing is significantly impaired because of the inhibition of cellular migration, a process crucial for tissue repair 50. Enhancing cellular migration is thus a viable strategy to expedite wound healing. Research has shown that BMSCs migrate to wound sites to increase the migratory activity of fibroblasts 51. Furthermore, the secretome from cells grown on 3D matrices has been demonstrated to enhance the proliferation and migration of fibroblasts, so as to improve wound healing in corneal transplant 52. Our previous study demonstrated that BMSCs cultured in 3D environments promote the migration of fibroblasts, which is a significant finding for advancing wound healing therapies 19. In this study, we employed a scratch assay to investigate whether BMSC@COPM enhanced fibroblast migration. Our results indicated that the BMSC@COPM group exhibited a significantly promoting migration ability compared to the other microsphere groups (P < 0.001) (Fig. 4A and 4C). These findings indicate that combining curcumin and OPM in BMSC@COPM increased fibroblast migration.

### BMSC-loaded microspheres enhance angiogenesis in HUVECs

Angiogenesis, driven by endothelial cells, play a pivotal role in the success of wound healing processes 53. To simulate the diabetic environment, such as under hypoxic 54 or high sugar conditions 55. Our experiment was conducted under high-sugar conditions. We assessed the angiogenic potential ability of BMSC@OPM via a tube formation assay. The BMSC@COPM group and the BMSC@OPM group presented significantly increased numbers of new blood vessels, especially for BMSC@COPM group (Fig. 4B and 4E). The cell survival rates across the microsphere groups subsequently significantly increased (*P* < 0.001) (Fig. 4D), with the BMSC@COPM group exhibiting the highest survival rate, as determined by an MTT assay. These results suggested that BMSC@COPM, with an open porous microsphere structure, significantly promoted the angiogenesis potential of HUVECs.

The regulation of wound microangiogenesis involves pathways closely associated with angiogenesis, with VEGF being intricately linked to HIF-1α accumulation. High glucose levels in diabetic wounds inhibit HIF-1α accumulation, leading to hypoxia and impaired cellular metabolism 56. VEGF is crucial for angiogenesis, tissue remodeling, cell proliferation, and vascular permeability 57. We found the protein expression of HIF-1α and VEGF in HUVECs were remarkably elevated by BMSC@OPM and BMSC@COPM than those of control under high-glucose conditions (P < 0.05, P < 0.01, P < 0.001) (Fig. 4F-I). The results revealed that both BMSC@OPM and BMSC@COPM successfully rescued the inhibition to HIF-1α and VEGF.

VEGF and PDGF regulate angiogenesis, re-epithelialization and tissue regeneration, which is important for wound healing 57, whereas FGF is another essential cell factor for angiogenesis and re-epithelialization 58. From Fig. 4J, we found the mRNA levels of PDGF, FGF, and VEGF in the four microsphere groups were elevated to varying degrees, with the BMSC@COPM group showing the most one (*P* < 0.05,* P* < 0.01, *P* < 0.001).

In conclusion, BMSC@COPM demonstrated superior lumen formation ability and elevated levels of proangiogenic factors. These results indicated that growing BMSCs on 3D spheres boosts their growth and the angiogenic potential of endothelial cells at wound site by elevating the release of angiogenic factors, which are consistent with earlier research 19. In this study, the novel microspheres utilized improved the survival environment of the material, and the combination of BMSCs and curcumin promoted the angiogenic ability, thereby promoting tissue regeneration.

### BMSC@COPM accelerates diabetic wound healing

**Figure 5:**
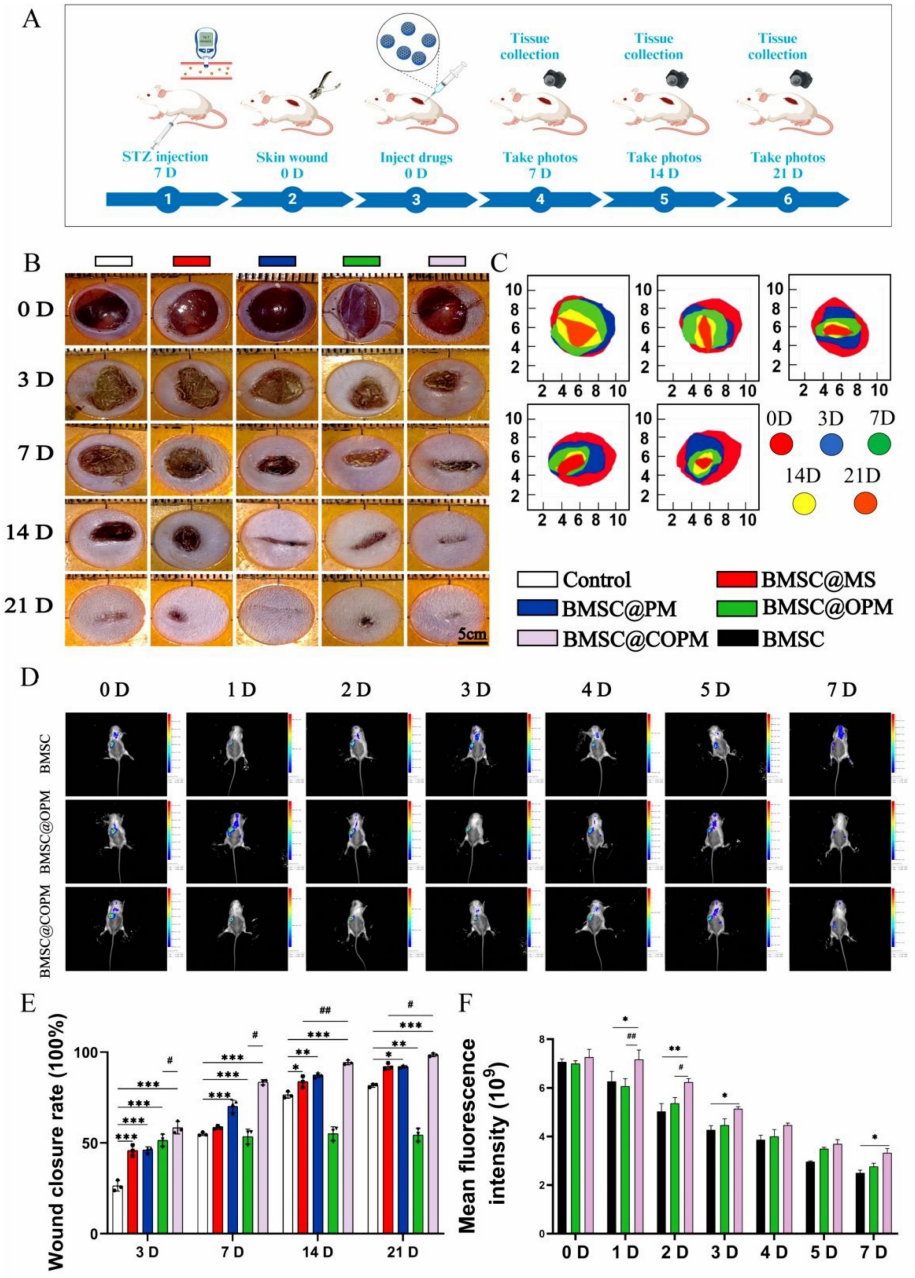
BMSC-loaded microspheres enhance the healing of diabetic wounds. (A) Flowchart of the animal experiments. (B) Images illustrating the wound healing progression in mice under specified treatment for 0, 3, 7, 14 or 21 days, respectively. (C) A fusion map of (B) in the same group at different times. (D) Representative images of live small animal images. (E) Quantitative analysis of (B). (F) Quantitative analysis of (D). *vs* the control group, ^*^P < 0.05, ^**^P < 0.01 and ^***^P < 0.001; *vs* the BMSC@COPM group, ^#^*P* < 0.05 and ^##^*P* < 0.01. The data shown in the figure are presented as the means ± SDs (n = 6).

Research conducted* in vitro* indicates that BMSC@COPM has the potential to be a wound dressing for incurable diabetic wounds. In order to avoid interference of the estrous cycle on metabolic indicators, this study utilized only male mice, which is consistent with previous reports 56,59,60. The diabetic animals were established as described previously. The fasting blood sugar value was greater than 16.7 mM, and both glucose and insulin tolerance were notably poorer compared to the normal group (Fig. S10). Experiments on animals were executed to evaluate the wound healing effects of the four microsphere groups following treatment (Fig. 5A). The tissue samples of all group (including Control, BMSC@MS, BMSC@PM, BMSC@OPM and BMSC@COPM) were collected on day 0, 3, 7, 14 and 21 (Fig. 5B, 5C). The groups treated with microspheres showed a notable decrease in wound area after 3 and 7 days (*P* < 0.001). Notably, the BMSC@COPM group exhibited superior wound healing efficacy, significantly outperforming the BMSC@OPM group (*P* < 0.05). It is pointed out that the BMSC@COPM group achieved the highest healing ratios of 82% and 97% on days 14 and 21, respectively, compared with only 75% closure in the control group on day 14 (Fig. 5E). These obvious results suggest that the synergistic reaction of curcumin and OPM in BMSC@COPM accelerated diabetic wound healing, which is consistent with the *in vitro* healing rate (Fig. S11). To determine BMSC colonization and survival rates. A small animal live imaging experiment was performed. By day 4, the fluorescence intensity of the BMSCs was maintained at 50%, but it swiftly disappeared on day 5. BMSC@COPM displayed the most intense fluorescence, which implies that BMSCs have a high level of colonization and survival in COPM (Fig. 5D and 5F).

### BMSC@COPM promotes diabetic wound remodeling

**Figure 6:**
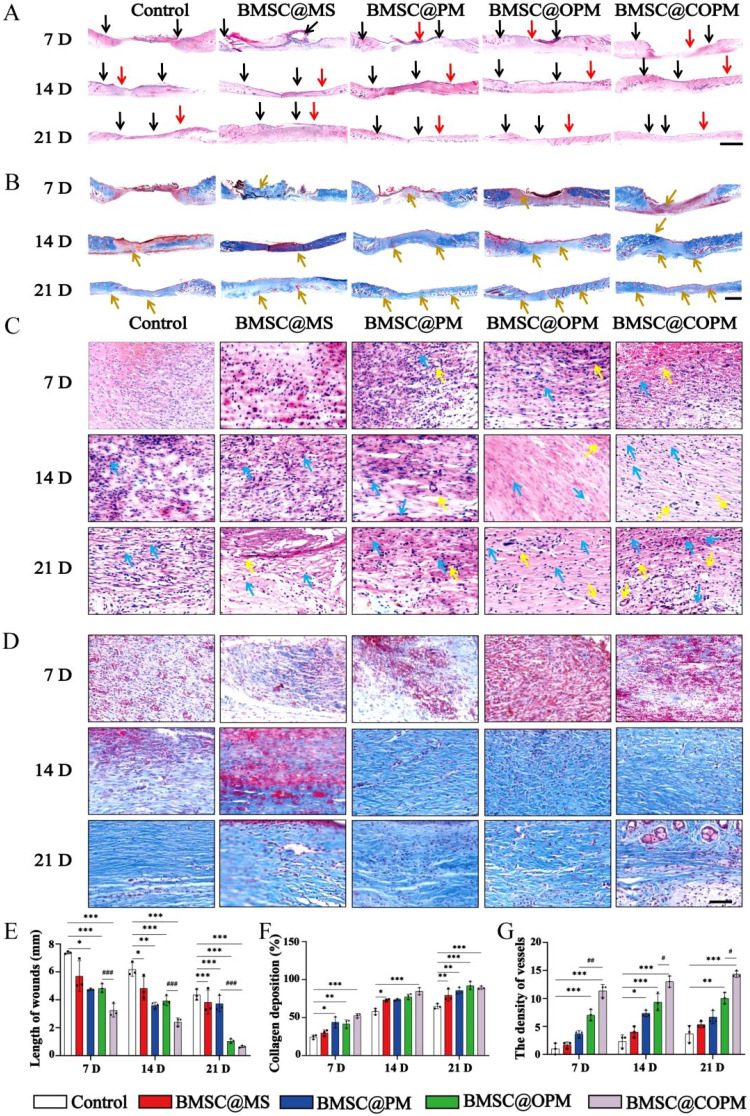
Examination of diabetic wounds treated with BMSC@COPM using histology. (A) H&E-stained panoramic images showing diabetic wounds in treated mice at days 7, 14 or 21, with the black arrows representing both ends of the wound (scale bars: 1 mm). (B) Representative panoramic images of Masson's trichome-stained wounds from treated diabetic mice at days 7, 14 or 21, with the the red arrows representing re-epithelialization and the orange arrows representing collagen deposition (scale bars: 1 mm). (C) HE-stained images (scale: 50 μm) were obtained at 7, 14 and 21 days after injury via high-power microscopy (the yellow arrows represent blood vessels; the blue arrows represent mature granulation tissues). (D) Masson's trichome staining images were obtained at 7, 14 and 21 days after injury under a high-power microscope (Scale bars: 50 μm). (E) Quantitative analysis of the wound area length in (A). (F) Analysis of collagen accumulation was conducted quantitatively at 7, 14, 21 days post-wounding. (G) New blood vessels and statistical analysis at 7, 14 and 21 days of skin wound healing. Compared with the control group, ^*^P < 0.05,^ **^P < 0.01 and ^***^P < 0.001; compared with the BMSC@COPM group, ^#^*P* < 0.05, ^##^*P* < 0.01 and ^###^*P* < 0.001. The data shown in the figure are presented as the means ± SDs (n = 6).

The contribution of the BMSC@COPM to tissue remodeling was assessed by monitoring wound length and collagen deposition at different stages of the postwounding. H&E staining confirmed the wound healing rates, with the BMSC@COPM group exhibiting the most rapid reduction in wound length, particularly during the initial and middle phases of healing (Fig. 6A and 6E). The statistical analysis showed that wound contraction occurred significantly faster in the BMSC@COPM group, particularly between day 7 and 14 (*P* < 0.05, *P* < 0.01, *P* < 0.001). We found that the wounds in the BMSC@COPM group were almost fully re-epithelialized, with reduced wound length and a significant presence of granulation tissue on the 21st day (Table 3). By day 7, the wounds in the BMSC@PM, BMSC@OPM, and BMSC@COPM groups had developed mature granulation tissue, with the BMSC@COPM group showing a rapid score increase until day 21. This represents good performance of an active healing process (Fig. 6C). In contrast, these healing parameters lagged behind those of the control group. Collectively, the result of H&E staining demonstrated the important effect of BMSC@COPM on accelerating the wound healing process, highlighting its potential as an effective therapeutic intervention for tissue remodeling in diabetic wounds.

Collagen is essential as a structural framework that facilitates angiogenesis, cellular migration, and regeneration of granulation tissue 61. Above all, essential collagen deposition and remodeling are crucial for increasing tissue tensile strength and facilitating effective healing 62. To determine the presence of newly produced collagen at the wound location, Masson's trichrome and picrosirius red stains were applied to the wounds at 7, 14, and 21 days (Fig. 6B, 6D, 6F and Table 3). The findings indicated that every BMSC-laden microsphere group showed a time-dependent rise in collagen production (P < 0.05, P < 0.01, P < 0.001), with the BMSC@COPM group demonstrating especially notable results. On day 14, the BMSC@COPM group developed abundant collagenation with a regular pattern and ideal network topology, which is indicated that the mature collagens were formed. On day 21, the BMSC@COPM group showed the most advanced maturation, marked by the emergence of new hair follicles and other skin appendages, indicating that the healed tissue closely resembled healthy skin and significantly improved healing outcomes.

Additionally, we analyzed the structural composition of the regenerating skin. During the healing process, the deposition of collagen increases 63. It was found that the ratio of type I collagen to type III collagen was increasing in mature skin tissue gradually 64. When comparing the impact of skin structure on wound healing at days 7 and 21, there was a notable rise in type I collagen deposition in both the BMSC@COPM and BMSC@OPM groups (Fig. S12A). Moreover, type III collagen deposition was the opposite (Fig. S12B and S12C). These results indicated that BMSC@OPM and BMSC@COPM facilitated efficient collagen remodeling and skin maturation. Throughout the entire development of diabetic wound healing, the therapeutic effect of BMSC@COPM is exceptionally remarkable.

### BMSC@COPM promotes angiogenesis during diabetic wound healing

**Figure 7:**
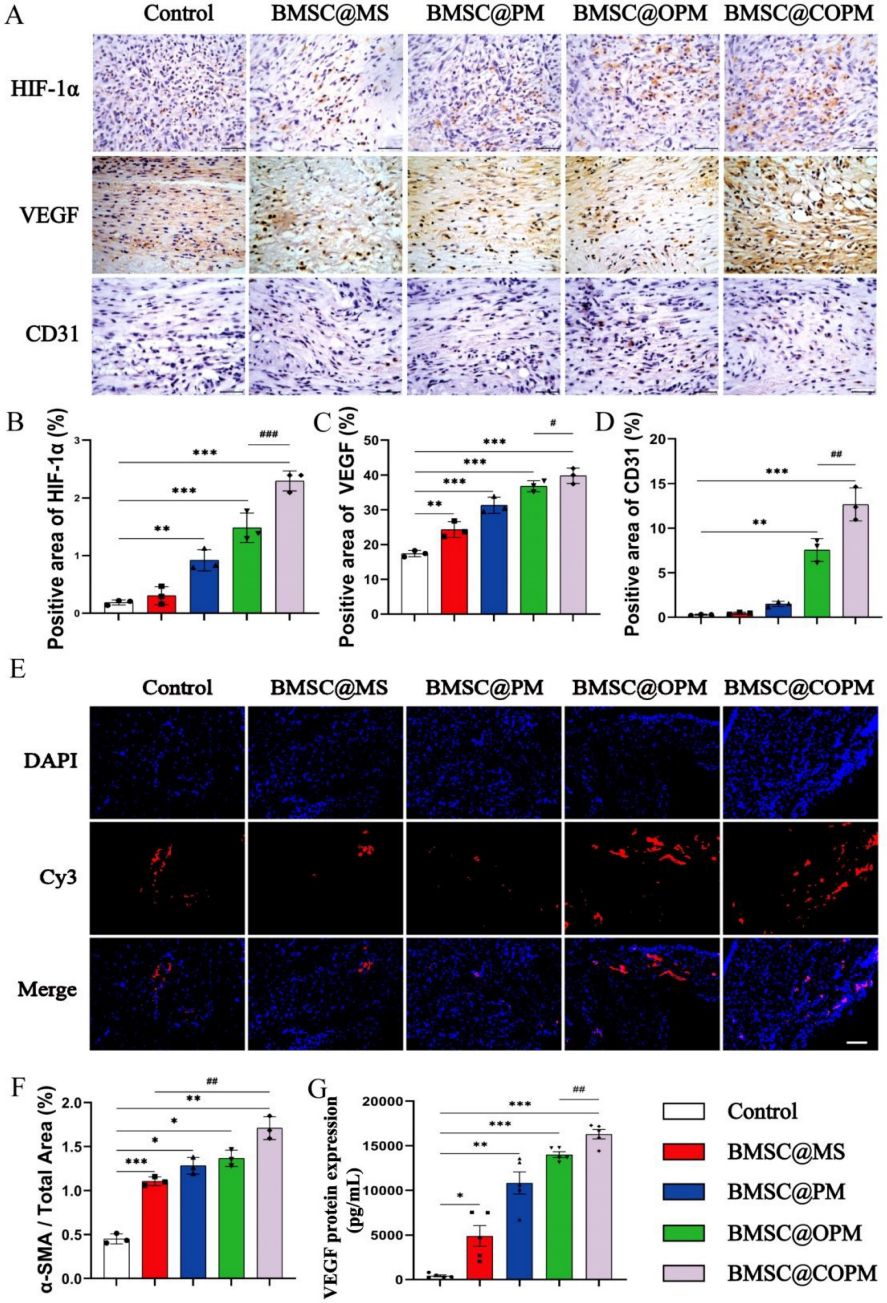
Effect of BMSC@COPM on angiogenesis in diabetic wounds. (A) Immunohistochemistry was performed on the 14th day of wound healing. (Scale bars: 50 μm). (B) The statistical analysis of HIF-1α protein expression in (A). (C) The statistical analysis of VEGF protein expression in (A). (D) The statistical analysis of CD31 protein expression in (A). (E) On day 14 post-wounding, α-SMA immunofluorescence was observed in sections of diabetic wounds (scale bars: 50 μm). (F) The statistical analysis of α-SMA protein expression in (E). (G) Measurement of VEGF protein levels in mice serum via ELISA. *vs* the control group, ^*^P < 0.05, ^**^P < 0.01, ^***^P < 0.001; *vs* the BMSC@COPM group, ^##^*P* < 0.01 and ^###^*P* < 0.001. The data shown in the figure are presented as the means ± SDs (n = 6).

Earlier research suggested that MSCs are ideal for boosting angiogenesis due to their capacity to increase the genes expression related to angiogenesis 65. We found that the angiogenic ability of BMSC@OPM and BMSC@COPM microsphere groups were improved at each posttreatment time point (*P* < 0.01, *P* < 0.001), with the BMSC@COPM group showing the most pronounced effects (Fig. 6C, 6G and Table 2). Hyperglycemia is known to suppress HIF-1α, thereby inhibiting wound healing. On the contrary, HIF-1α promote angiogenesis could enhance neovascularization 66. In our research, HIF-1α expression determined through immunohistochemical staining, was notably induced by different biological material treatment group, particularly in the BMSC@COPM mice (Fig. 7A and 7B). The combined application of BMSCs and curcumin-loaded OPM notably improved the hypoxic state of diabetic wounds.

VEGF regulate the development of new blood vessels, in addition, angiogenesis is strongly linked to CD31+ endothelial cells and α-SMA+ smooth muscle cells 67,68. The expression of VEGF and CD31 was significantly increased in all four BMSC-laden microsphere groups, with the BMSC@COPM group showing the most significant increase (*P* < 0.01, *P* < 0.001; Fig. 7A, 7C, 7D and 7G). The expression of α-SMA was similar (Fig. 7E and 7F). In diabetic wound tissue, the reduced levels of α-SMA, VEGF, and CD31 were linked to impaired angiogenesis, which was improved in the BMSC@COPM group. These findings confirmed the suitable microstructures of the cell carrier produced by the BMSC@COPM, and the synergistic effect of curcumin and OPM was beneficial for enhancing epithelial regeneration and new blood vessel formation. When HIF-1α binds to the hypoxia response element (HRE), the VEGF gene promoter was activated. In diabetic wound tissue, decreased α-SMA, VEGF, and CD31 levels were associated with impaired angiogenesis, which was enhanced in the BMSC@COPM group 69,70. In this research, we found that HIF-1α expression decreased under high-glucose conditions, along with a notable reduction in VEGF levels. BMSC@COPM intervention reversed these changes in protein expression, ultimately leading to the promotion of angiogenesis. These findings suggest that the mechanism by which HIF-1α promotes angiogenesis is associated with the activation of VEGF.

### BMSC@COPM promoted the polarization of macrophages from the M1 to the M2 phenotype

**Figure 8:**
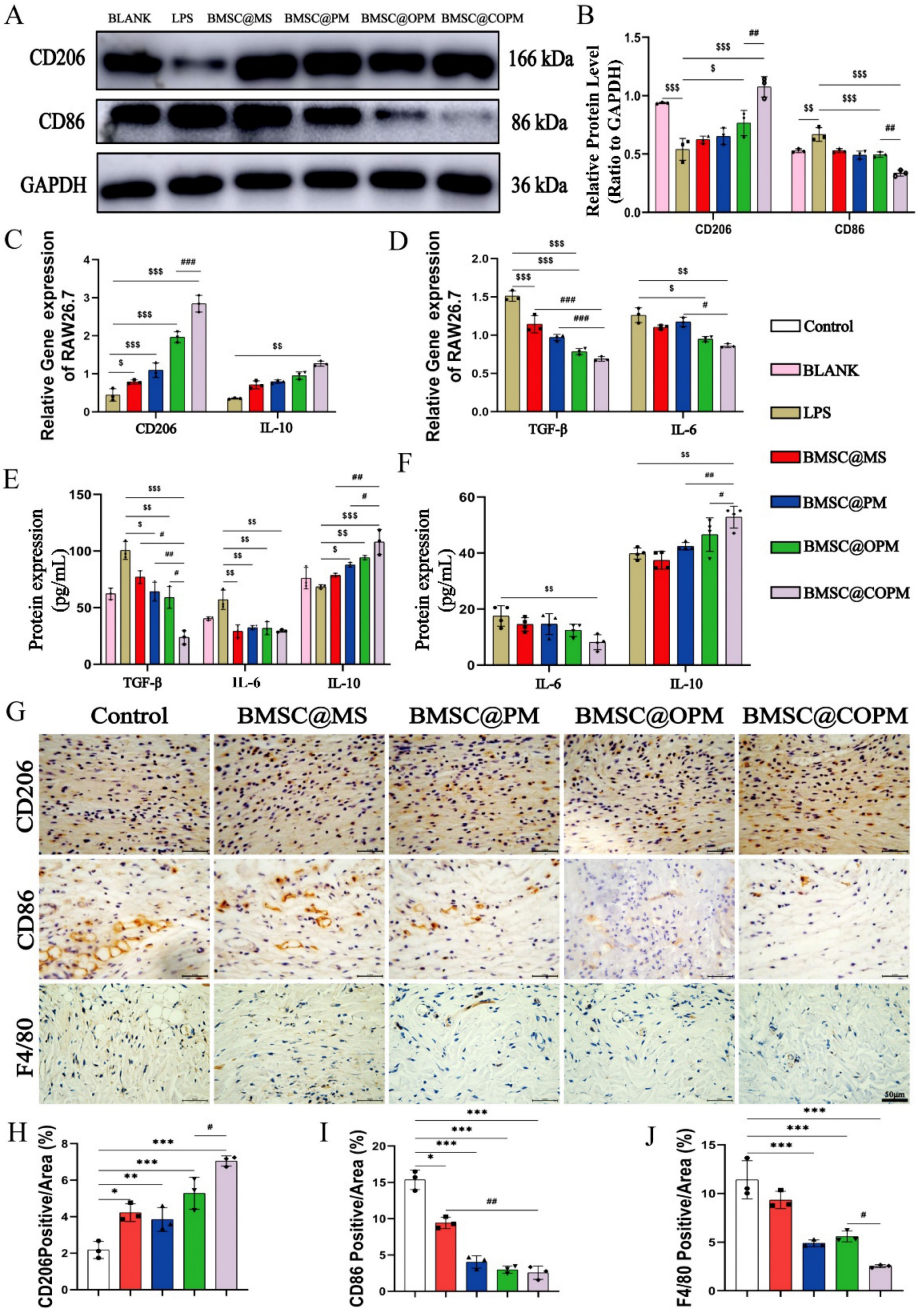
Effect of BMSC@COPM on macrophage polarization. (A) Images representing blotting of CD206 and CD86 in macrophages was evaluated by Western blotting. (B) Statistical analysis of CD206 and CD86 expression. (C) The relative level of anti-inflammatory-related genes, which was detected via qRT‒PCR. (D) The relative level of proinflammatory-related genes was evaluated via qRT‒PCR. (E) Proinflammatory and anti-inflammatory protein expression in the RAW264.7 macrophage supernatant was evaluated via ELISA. (F) Proinflammatory and anti-inflammatory protein expression in mouse serum was evaluated by ELISA. (G) Images representing F4/80, CD86 and CD206 protein in the diabetic animal wound after treated for 14 days via IHC (scale bar: 50 μm). (H, I and J) Statistical analysis of F4/80, CD86 and CD206 expression. Compared with the control group, ^*^P < 0.05, ^**^P < 0.01 and ^***^P < 0.001; compared with the BMSC@COPM group,^ #^*P* < 0.05, ^##^*P* < 0.01; compared with the LPS group, ^$^*P* < 0.05,^ $$^*P* < 0.01, and ^$$$^*P* < 0.001. The data shown in the figure are presented as the means ± SDs (n = 3).

Macrophages, derived from the immune system, are crucial in tissue repair, especially during the inflammation stage. Excessive activation of proinflammatory M1 macrophages is the main factor of delayed diabetic wound healing 71. *In vivo*, macrophages undergo a polarization shift from the M0 to the M1 state 72-74, with M1 macrophages demonstrating robust pathogen and cellular debris clearance capabilities 75. Known for their production of proinflammatory cytokines and markers such as CD86 76, these M1 macrophages are key mediators of the initial inflammatory response. Transitioning from the M1 to the M2 phenotype is crucial for moving through the inflammation phase and aiding later repair stages 77,78. Activated M2 macrophages counteract inflammation by secreting anti-inflammatory cytokines and markers (such as CD206) 79, making CD206 and CD86 pivotal biomarkers for assessing macrophage polarization 80. To investigate the synergistic influence of BMSCs and curcumin within the BMSC@COPM construct on macrophage polarization, we simulated an *in vitro* inflammatory scenario by inducing macrophages with LPS 81,82. The anti-inflammatory impact of the BMSC@COPM was evaluated via Western blot analysis. Following LPS induction, CD206 expression was reduced and CD86 expression was opposite (*P* < 0.01), suggesting the shift of M0 macrophages to the M1 phenotype. In contrast, both the BMSC@OPM and the BMSC@COPM treatment reversed these changes, especially for BMSC@COPM (*P* < 0.001) (Fig. 8A and 8B). The results indicate that BMSC@COPM promoted the polarization of RAW 264.7 macrophages from the M1 to the M2 phenotype.

The expression profiles of common inflammation-associated genes in RAW264.7 macrophages following LPS stimulation were also analyzed 83. In the treatment groups, the gene expression levels of the inflammatory markers (TGF-β, IL-6) were notably reduced compared to the control group, while the anti-inflammatory markers (CD206, IL-10) were the opposite (Fig. 8C and 8D). The change trend of of IL-6 and IL-10 protein level were consistent with their gene expression respectively (Fig. 8E and 8F). The results revealed that the combined application of BMSCs and curcumin in BMSC@COPM can effectively suppress the production of proinflammatory cytokines, through inducing RAW 264.7 cell polarization toward an anti-inflammatory (M2) phenotype 84. Excessive inflammation leads to increased secretion of proinflammatory cytokines, complicating their polarization into reparative M2 phenotypes and perpetuating chronic inflammation that delays wound healing 85,86. Immunohistochemical staining was conducted on days 7 and 14 to investigate macrophage infiltration in diabetic wound tissue. (Fig. 8G). In the BMSC-laden microsphere groups, especially the BMSC@COPM group, there was an increase in CD206 expression levels. On day 7, the treatment groups showed a variably lower CD86 level compared to without treatment (Fig. 8H and 8I). The finding *in vivo* was consistent with the results* in vitro*. F4/80 is a specific macrophage marker consistently expressed across various immune or inflammatory models 87,88. As shown in Fig. 8J, F4/80 level was notably lower in the microsphere groups containing BMSCs, particularly in the BMSC@COPM group (on day 14 postwounding). These outcomes imply that there is a higher presence of M1 macrophages in untreated diabetic wounds, which prolongs the inflammation period. By day 14, treatment with BMSC@COPM led to a notable promotion to M2 macrophages accompanied by a decrease in F4/80 expression, suggesting a significant reduction in inflammatory infiltration. This evidence suggests that BMSC@COPM facilitates diabetic wound healing by inducing macrophages polarization toward M2, and accelerating the transition from the delayed inflammatory phase to the skin tissue repair phase. It was reported that M2 polarization via PI3K/Akt 89 or AMPK 90-92 enhances tissue repair via mechanisms that increase cell proliferation, promote angiogenesis, and depress inflammation. Our observations of the use of the BMSC@COPM treatment for diabetic wounds align with these reported mechanisms, suggesting potential pathway involvement.

### BMSC@COPM stimulates autophagy to promote the migration and proliferation of NIH-3T3 cells

**Figure 9:**
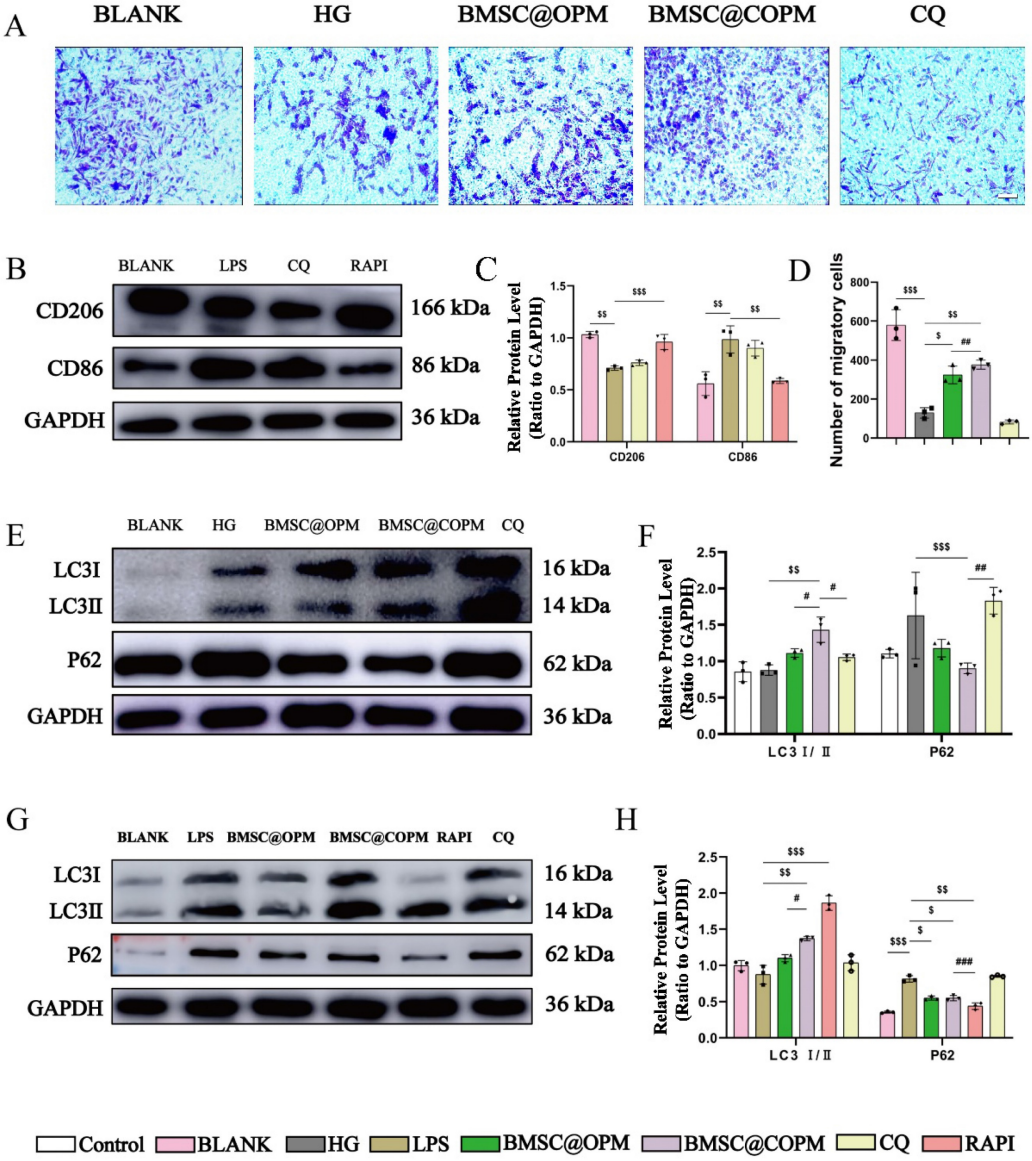
Autophagy induction by BMSC@COPM enhances macrophage polarization and promotes migration and proliferation. (A) Transwell assays of NIH-3T3 cells were observed under a microscope, which were treated with BMSC@COPM in a high-glucose environment (Scale bar: 100 μm). (B) Representative WB blot of CD206 and CD86 in RAW 264.7 cells after were treated with LPS, rapamycin or chloroquine, respectively. (C) Quantitative analysis of CD206 and CD86 protein expression in (B). (D) Quantification of the transwell assay results. (E) Representative images of LC3-II and P62 protein blots in NIH-3T3 cells treated with HG and then treated with CQ (10 μM) or BMSC@COPM. (F) Statistical analysis for P62 and the LC3-II/LC3-I ratio in (E). (G) Representative images of LC3-I/II and P62 protein blots in RAW 264.7 cells, which were induced with LPS and then treated with 10 μM rapamycin (an autophagy inducer, RAPA), 10 μM CQ, or BMSC@COPM. (H) Statistical analysis protein expression related to autophagyin. *vs* the control group, ^**^P < 0.01 and ^***^P < 0.001; *vs* the LPS group and HG group, ^$^*P* < 0.05,^ $$^*P* < 0.01 and ^$$$^*P* < 0.001; *vs* the BMSC@COPM group, ^#^*P* < 0.05, ^##^*P* < 0.01, ^###^*P* < 0.001. The data shown in the figure are presented as the means ± SDs (n = 3).

In cellular biology, autophagy is recognized for its effect on facilitating cell migration and proliferation 93,94. The research explored whether BMSC@COPM might perform the same function as the report. To elucidate this relationship, we employed a transwell assay (Fig. 9A and 9D). We cultured NIH-3T3 cells in a high-glucose environment for 36 hours, then added BMSC@COPM for another 24 hours of incubation. In parallel experiments, chloroquine (an established autophagy inhibitor, CQ) was selected for combination with BMSC@COPM treatment (Fig. S13). Our research shows that high-glucose environments greatly hinder the growth and movement of NIH-3T3 cells. However, adding BMSC@COPM reversed this inhibition, leading to a marked increase in both cellular activities. Conversely, the application of CQ negated these effects, suggesting that the observed enhancements are contingent upon autophagy activation. These results underscore the inhibitory impact of sustained exposure to high glucose levels on cellular autophagy, which hinders cell migration and proliferation. Nevertheless, supplementation with BMSC@COPM appeared to counteract these effects by promoting autophagy and hence improving cell proliferation and migration.

P62 and the LC3I/II ratio are pivotal markers for assessing autophagy 95. P62 is a key receptor in autophagy processes, and it accumulates within the cell when autophagy is compromised 96. Conversely, the quantity of LC3-II is an indicator of the number of autophagosomes, with its expression level reflecting autophagy activity 97. The data indicated a marked elevation in P62 expression in both the high-glucose condition and chloroquine treatment relative to the control. This observation, coupled with the lack of a substantial increase in LC3 expression, points toward the suppression of autophagy under chronic high-glucose conditions. Significantly, BMSC@COPM treatment increased LC3 II expression and decreased P62, indicating its effectiveness in promoting autophagy. Overall, these results revealed that BMSC@COPM plays a crucial role in enhancing the growth and movement of NIH-3T3 cells by triggering autophagy (Fig. 9E and 9F).

### Autophagy induction by BMSC@COPM enhances macrophage polarization toward the M2 phenotype

BMSC@COPM has been shown to encourage macrophages to adopt an anti-inflammatory phenotype. A prior investigation revealed that poly(L-lactic acid) nanofibrous membranes with immunomodulatory properties aid in diabetic wound healing by stimulating macrophage autophagy 83. We further investigated whether this polarization-promoting effect is related to the activation of macrophage autophagy 79. We have assessed the impact of modulating autophagy on the polarization of macrophages after LPS induction, and detected the expression levels of markers characteristic of M1 or M2 macrophages to quantify the phenotypic shift induced by alterations in autophagy levels. LPS-induced macrophages were treated with RAPA or CQ. The RAPA group, which served as a positive control, demonstrated activated autophagy, whereas the CQ group (negative control) exhibited suppressed autophagy. Our results showed that the RAPA group had notably higher CD206 expression and significantly lower CD86 expression. In contrast, the CD206 and CD86 levels in the CQ group remained relatively stable. These findings imply that triggering autophagy can successfully promote the macrophages polarization from the proinflammatory M1 state to the anti-inflammatory M2 state, which may improve the wound healing process (Fig. 9B and 9C).

To assess how BMSC@COPM affects macrophage autophagy, BMSC@COPM was added to RAW264.7 macrophages induced by LPS (Fig. 9G and 9H). Compared with the LPS group, both the BMSC@OPM and BMSC@COPM groups presented significant upregulation of LC3II and substantial downregulation of P62. Notably, the BMSC@COPM group presented pronounced increases in LC3II expression and decreases in P62 expression. These results demonstrated that LPS induction polarized macrophages toward the M1 phenotype and concurrently suppressed autophagy, impeding complete macrophage polarization. However, the addition of the BMSC@COPM activated autophagy and increased the number of M1 macrophages that polarized to the M2 type. Future research will focus more on developing targeted nanomaterials for therapeutic applications 98.

### Autophagy induction by a nonclassical mechanism dependent on PI3K/Akt/mTOR enhances macrophage polarization

**Figure 10:**
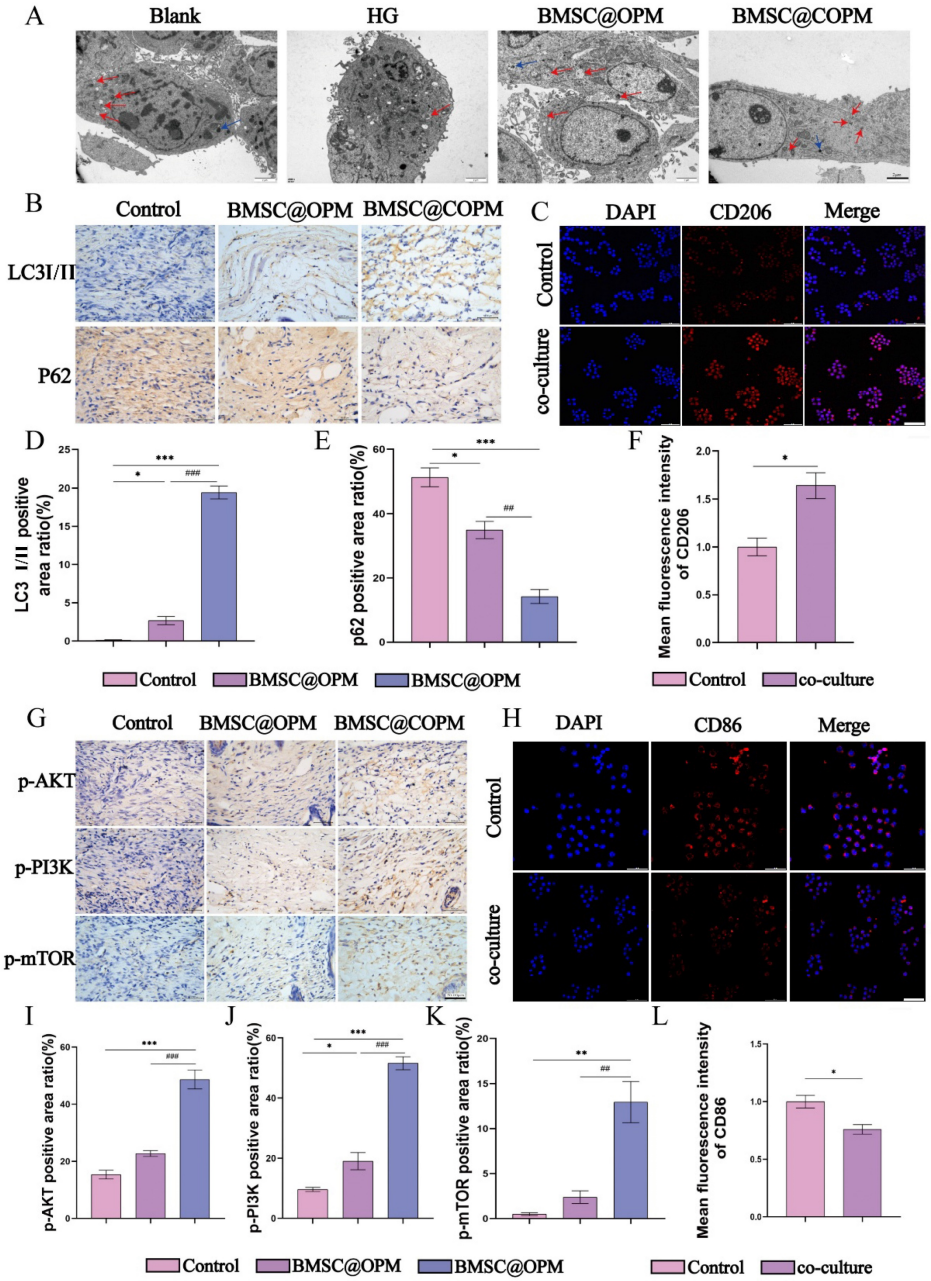
The relationship between autophagy and the PI3K/Akt/mTOR signaling pathway. (A) TEM detected autophagosomes in NIH-3T3 cells exposed to BMSC@COPM under high glucose conditions (Scale bar: 2 μm, the red arrows represent autophagosomes). (B) Representative immunohistochemical images of LC3-I/II and P62 in different animal (50 μM). (C) Representative images of CD206 immunofluorescence in RAW264.7 macrophages after coculture with BMSCs. (D) Quantification analysis of protein expression of LC3-I/II. (E) Statistical analysis for p62 protein expression. (F) Quantification of CD206 protein expression. (G) Representative immunohistochemical images of p-Akt, p-PI3K and p-mTOR in different groups of mice (50 μM). (H) Representative images of CD86 immunofluorescence in RAW264.7 macrophages after coculture with BMSCs. (I-K) Statistical analysis for protein levels of p-Akt, p-PI3K, p-mTOR in (G). (L) Quantification analysis of CD86 protein expression in (H). *vs* the control group, ^*^P < 0.001, ^**^P < 0.01 and ^***^P < 0.001; *vs* the BMSC@COPM group*, ^##^P* < 0.01, ^###^*P* < 0.001. The data shown in the figure are presented as the means ± SDs (n = 3).

BMSCs release a range of cytokines, which aids in tissue repair and regeneration. Curcumin possesses several biological properties, including anti-inflammatory, anti-oxidant, and immunomodulatory effects 13,14. Curcumin regulates the paracrine activity of BMSCs, promoting their proliferation and the secretion of more cytokines 99. We found that this synergistic mechanism is related to autophagy. Figure 10A shows that few autophagosomes in the high-glucose condition compare to normal group. BMSC@OPM and BMSC@COPM reversed the inhibition of autophagy. BMSC@COPM and BMSC@OPM activate autophagy protein expression in animal skin tissues, and these results supported the conclusion *in vitro* (Figure 10B, D, E). Research reports indicate that autophagy can lead to the downregulation of PI3K/Akt/mTOR and promote tissue repair 100, which is the classic pathway by which PI3K/Akt/mTOR inhibits autophagy. The study explores whether autophagy induced by PI3K/Akt/mTOR pathway in wound healing. Research indicates that enhancing the PI3K/Akt/mTOR aids in skin regeneration 101,102. The PI3K/Akt/mTOR pathway enhances angiogenesis and encourages the growth and movement of fibroblasts 103-105. This reflects the complexity of network regulation, a nonclassical dependence on PI3K/Akt/mTOR to promote autophagy. This study focused on 21-day diabetic mice during the proliferation period of wound repair. Figures 10G and 10I-K illustrate that BMSC@COPM and BMSC@OPM promoted the protein expression of p-AKT, p-PI3K and p-mTOR. Moreover, the protein level of P62 decreased. BMSC@COPM and BMSC@OPM promote angiogenesis, fibroblast proliferation and migration by activating PI3K/Akt/mTOR, thereby facilitating wound healing. Moreover, appropriate activation of autophagy is conducive to high-quality repair. These results indicate that BMSC@COPM induces autophagy in a nonclassical PI3K/Akt/mTOR-dependent manner. The results of coculture of RAW264.7 macrophages with BMSCs indicated that cell crosstalk effected macrophage polarization (Figure 10C, F, H, L). This combined effect enhances diabetic wound healing, decreases inflammation, and encourages tissue regeneration. Moreover, we considered the different biological functions of simple combinations of curcumin, BMSC and OPM with BMSC@COPM. We found that the ability of the BMSC@COPM combination was better than that of simple combinations and promoted the migration ability of fibroblasts (Fig. S14).

BMSCs have the ability to renew themselves, differentiate into multiple cell types, and secrete a range of cytokines, all of which aid in tissue repair and regeneration 106. Research found that BMSCs improve the wound healing environment by secreting anti-inflammatory factors, promoting angiogenesis, and regulating immune responses 107. Curcumin, a natural polyphenol, has various biological functions such as antioxidant, anti-inflammatory, antibacterial, and immunomodulatory properties. In diabetic wounds, curcumin reduces oxidative stress, inhibits inflammatory responses, and promotes wound healing 108. The current study demonstrated that curcumin enhances the activity and function of BMSCs, promoting their proliferation and increasing their cytokine secretion. This combined effect decreases inflammation and fibrosis, and encourages tissue regeneration. The mechanism underlying the ability of curcumin to promote BMSC proliferation may be regulated via activing autophagy 19. Earlier research indicates that curcumin stimulates the PI3K/AKT/Nrf2, which enhances the proliferation and differentiation capabilities of stem cells 99. Curcumin also regulates the paracrine activity of BMSCs, increasing both the types and quantities of cytokines they secrete. For example, curcumin can induce BMSCs secrete abundant anti-inflammatory factors and angiogenesis factors, thereby improving the wound healing environment 42. The results indicate that curcumin can improve the therapeutic effectiveness of BMSCs in healing diabetic wounds through various mechanisms.

In the context of clinical applications, stem cell therapy has demonstrated substantial potential for treating diabetic wounds by effectively promoting tissue regeneration 109. However, a critical challenge in stem cell therapy is maintaining cellular activity and stemness. Various carriers have been designed to increase the therapeutic strategy of stem cells. Among these, porous PLGA microspheres, a class of biocompatible and absorbable microspheres, have been widely utilized as delivery vehicles for drugs or cells in clinical settings 110. Consequently, the integration of PLGA microspheres with stem cells represents a novel strategy for addressing traditionally intractable diseases.

Compared with conventional growth factor therapy, porous microspheres combined with stem cell therapy, which were prepared via gas-assisted volatile micro-emulsification technology in this study, offer several advantages. They can regulate immunity, promote extracellular matrix remodeling, and facilitate vascular and neural regeneration, thereby showing promise for clinical application. However, this approach also faces product price, stability and transportation challenges. Additionally, addressing ethical issues is crucial to ensure that *in vivo* stem cell applications comply with market regulations.

Taken together, our work presents a significantly distinct and innovative material strategy compared with the literature on porous microspheres. While recent publications have utilized materials such as gelatin hydrogels, PLGA/gelatin composites, PLLA/PLGA/collagen, sodium alginate/polyacrylamide, or TiO_2_/MgO, our microspheres are uniquely fabricated from PLGA via a gas-assisted volatilization microemulsion technique. This results in highly controllable porous structures with a distinctively rough surface morphology, which is crucial for enhanced cell adhesion, infiltration, and proliferation. More fundamentally, our innovation lies in functional integration: we loaded these optimized PLGA porous microspheres with curcumin@BSA nanoparticles and BMSCs. According to the literature, adding curcumin to stem cell transplantation enhances biological function outcomes 111-113. Our experiments showed that BMSCs activity was enhanced when curcumin was present. This synergistic combination of nanomedicine (precise drug delivery) and cellular therapy within a uniquely structured porous carrier is a key differentiator 114-116. This synergistic combination is better than a simple mixture. We initially conducted a pre-experiment with a mixed sample of microspheres, curcumin, and bone marrow stem cells as controls. We found that the ability of the simple mixed sample to promote fibroblast proliferation was not as obvious as that of the BMSC@COPM group (Figure S14). Unlike studies focusing on single functionalities (e.g., drug release, mechanical filling, or specific antioxidant properties), our material actively regulates the wound microenvironment through controlled curcumin release and BMSC activity. We demonstrated novel mechanisms, such as the activation of autophagy to alleviate inflammation and promote vascularization/collagen regeneration. This integrated approach combining advanced material fabrication (novel structure), nanodrug loading (enhanced hydrophobic drug capacity) and stem cell delivery for multifaceted diabetic wound healing represents a substantial functional and structural innovation beyond the scope of prior porous microsphere research.

## Conclusion

In the present investigation, we developed an innovative injectable, open-pore microsphere system using PLGA as a substrate, which encapsulates curcumin (denoted as COPM). These microspheres, characterized by their porous architecture and drug-encapsulation capabilities, effectively facilitated the adhesion and proliferation of BMSCs. The composite system, BMSC@COPM, demonstrated a sustained release profile of both curcumin and BMSCs, ensuring their secure anchorage to the wound's basal layer and fostering robust tissue regeneration. Our results emphasize the diverse therapeutic possibilities of BMSC@COPM. BMSC@COPM notably boosted the growth and movement of fibroblasts, essential for tissue healing. Moreover, it enhanced the ability of human umbilical vein endothelial cells (HUVECs) to promote angiogenesis, which is essential for vascularization. BMSC@COPM also reduced chronic inflammation and sped up the healing of diabetic wounds. Collectively, these results underscore the therapeutic potential of BMSC@COPM in promoting wound healing, enhancing tissue remodeling, stimulating neovascularization, and eliciting anti-inflammatory responses. BMSC@COPM, an injectable microcarrier combining stem cells and therapeutic agents, shows potential for improving the treatment of diabetic ulcers.

## Supplementary Material

Supplementary figures.

## Figures and Tables

**Table 1:** Compared with other advanced functional microsphere designs, highly porous microspheres have structural and functional advantages.

Different microspheres	The structural advantages of porous microspheres compared to other materials	The functional advantages of porous microspheres compared to other materials
Polymer self-assembled microspheres 23	Porous microspheres possess controllable microporous channels capable of effectively loading nanoparticles and drugs. In contrast, polymer self-assembly systems lack structurally controllable porous pore structures.	Porous microspheres can load multiple cell types and drugs to modulate the tissue microenvironment. In contrast, polymer self-assembly systems demonstrate low drug release efficiency and limited cell loading and protection capacity.
Probiotic-loaded microspheres 24	Porous microspheres exhibit an interconnected pore structure and can effectively load drug nanoparticles. In contrast, probiotic carrier formulations typically employ complex emulsion structures to protect probiotics.	Porous microspheres can load stem cells and drugs to modulate the tissue microenvironment, demonstrating the ability for sustained-release drug delivery. In contrast, probiotic formulations rely on specialized protective structures to exert their physiological activity.
Growth factor-eluting systems 25	Porous microspheres can effectively promote the slow release of curcumin and enhance wound repair. In contrast, the sustained-release system for bioactive factors focuses more on achieving low-dose, long-acting drug release.	Porous microspheres maintain stem cell activity and promote their proliferation and differentiation. In contrast, growth factor sustained-release systems focus more on modulating the local microenvironment.

**Table 2:** Sequences of the primers

Name of primer	Primer sequence
M-TGF-β-F	AGACGGAATACAGGGCTTTCGATTCA
M-TGF-β-R	CTTGGGCTTGCGACCCACGTAGTA
M-IL10-F	GCTCTTACTGACTGGCATGAG
M-IL10-R	CGCAGCTCTAGGAGCATGTG
M-CD206-F	CCTATGAAAATTGGGCTTACGG
M-CD206-R	CTGACAAATCCAGTTGTTGAGG
M-IL-6-F	ACCAGAGGAAATTTTCAATAGGC
M-IL-6-R	TGATGCACTTGCAGAAAACA
H-VEGF-F	TCCTCACCCGATAATGGTGGA
H-VEGF-R	CCAGGAAAGCAATCACATTCCC
H-PDGFbb-F	GCACACGCATGACAAGACGGC
H-PDGFbb-R	AGGCAGGCTATGCTGAGAGGTCC
H-FGF21-F	CTGCAGCTGAAAGCCTTGAAGC
H-FGF21-R	GTATCCGTCCTCAAGAAGCAGC
M-β-actin-F	CCAAGGCCAACCGCGAGAAGATAGC
β-actin-R H-β-actin-F H-β-actin-R	AGGGTACATGGTGGTGCCGCCAGAC CATGTACGTTGCTATCCAGGC CTCCTTAATGTCACGCACGAT

**Table 3:** Histopathological scoring for wound assessment

Groups	Collagen deposition	Mature granulation tissue	Re-epithelialization	Blood vessels	Total
Day 7					
Control	0	0	0	0	0
BMSC@MS	1	0	0	0	1
BMSC@PM	1	1	1	1	4
BMSC@OPM	1	1	1	1	4
BMSC@COPM	1	1	1	1	4
Day 14					
Control	1	1	3	0	5
BMSC@MS	1	2	4	0	7
BMSC@PM	2	2	4	1	9
BMSC@OPM	2	2	4	1	9
BMSC@COPM	3	3	4	2	12
Day 21					
Control	2	2	4	0	8
BMSC@MS	2	2	4	1	9
BMSC@PM	3	2	4	1	10
BMSC@OPM	3	3	4	2	12
BMSC@COPM	3	4	4	3	14

## Data Availability

The corresponding author can provide the data supporting this study's findings upon a reasonable request.
