# Supplementary material for: Open Porous Microenvironment-regulatory Microspheres Loaded with Curcumin@BSA NPs/BMSCs for Diabetic Wound Treatment
Source: Theranostics. 2026 Feb 11;16(9):4521–50. doi: 10.7150/thno.120285 (PMC12963991; doi:10.7150/thno.120285)
Supplement: Supplementary file 1 — Supplementary figures. [file thnov16p4521s1.pdf]

Open Porous Microenvironment-regulatory Microspheres Loaded with Curcumin@BSA NPs/BMSCs for Diabetic Wound Treatment

Zhe Liu[a, 1], Qinzhou Zheng[b, 1], Dong Zhou[c], Anqi Lin[c], Lan Xiao[g], Haifeng Liu[b], Keqin Ji[e, f], Huifen Qiang[e, f], Xinxin Sui[b], Yulin Li[c], Yan Wu[b,*], Jie Gao[e, f,**], Lan Liao[a,d,h,***], Xiaohuan Yuan[b, ****]

[a] School of Stomatology, Jiangxi Medical College, Jiangxi Province Key Laboratory of Oral Biomedicine, Jiangxi Province Clinical Research Center for Oral Diseases, Nanchang University, Nanchang 330006, China.

[b] College of Life Science, Mudanjiang Medical University, Mudanjiang 157011, China.

[c] Engineering Research Centre for Biomedical Materials of Ministry of Education, Frontiers Science Center for Materiobiology and Dynamic Chemistry, School of Materials Science and Engineering, East China University of Science and Technology, Shanghai 200237, China.

[d] The First Affiliated Hospital, Jiangxi Medical College, Nanchang University, Nanchang 330006, China.

[e] Changhai Clinical Research Unit, Shanghai Changhai Hospital, Naval Medical University, Shanghai 200433, China.

[f] Shanghai Key Laboratory of Nautical Medicine and Translation of Drugs and Medical Devices, Shanghai 200433, China.

[g] School of Medicine and Dentistry, Griffith University, QLD 4222, Australia.

[h] Jinggangshan University, Ji'an, Jiangxi, 343009, China.

[1] These authors contributed equally to this work

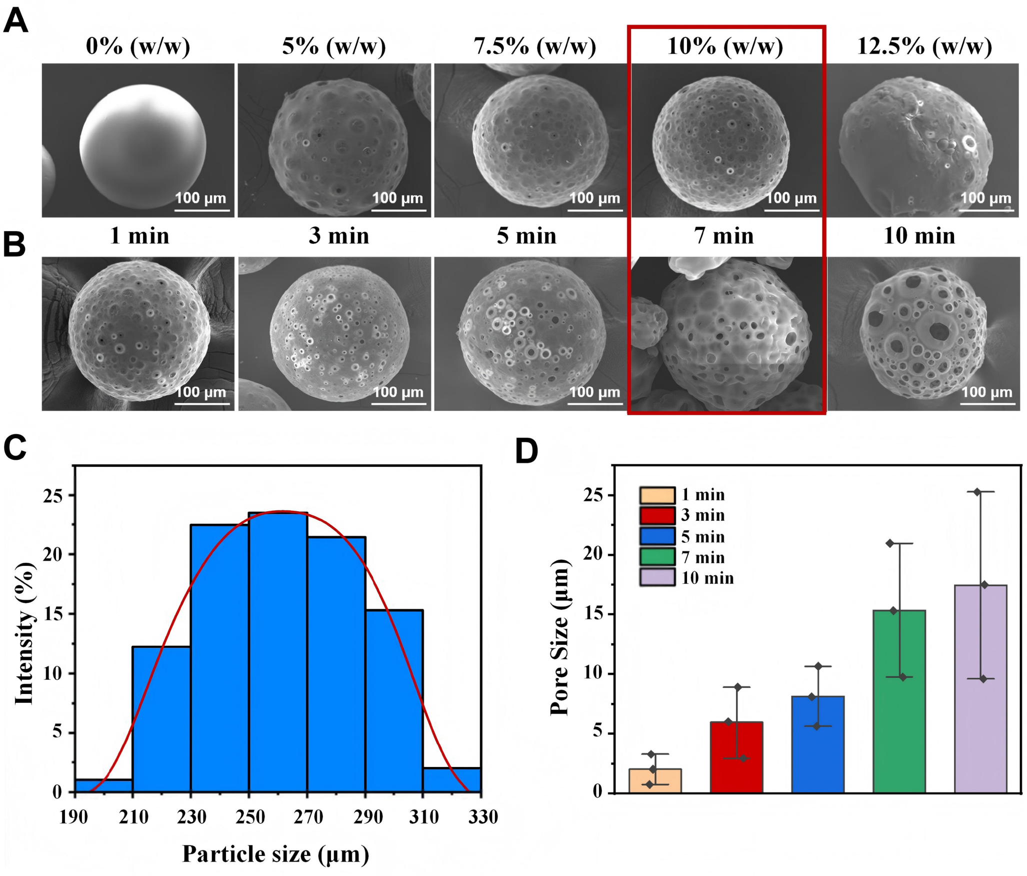

Fig S1. Surface morphology of microspheres synthesized with (A) different NH_4HCO_3 concentrations (a) and (B) different surface-alkalization-treatment times (b). (C) Size distribution of the microspheres. (D) Pore size distribution on the surface of the microspheres after surface-alkali treatment for 1 min, 3 min, 7 min, 10 min and 15 min.

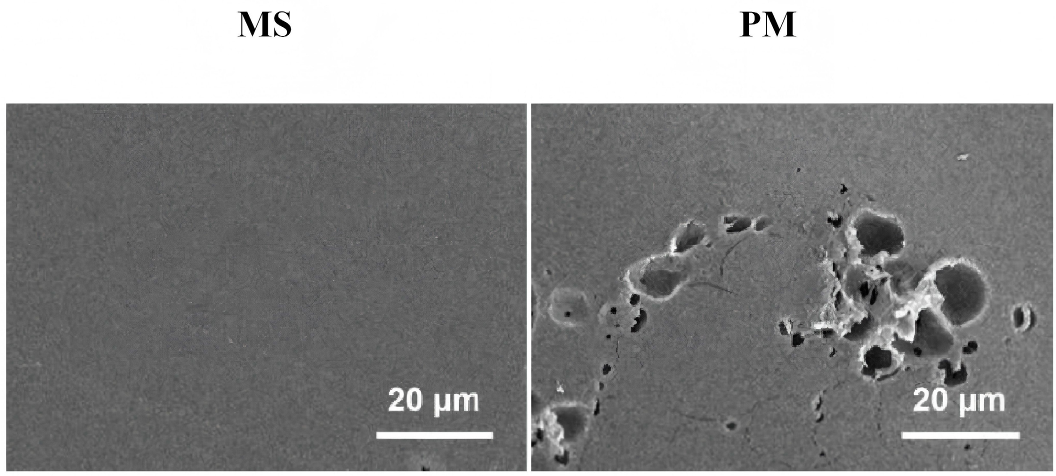

Fig S2. The internal section morphology of the PLGA MS, PM and OPM.

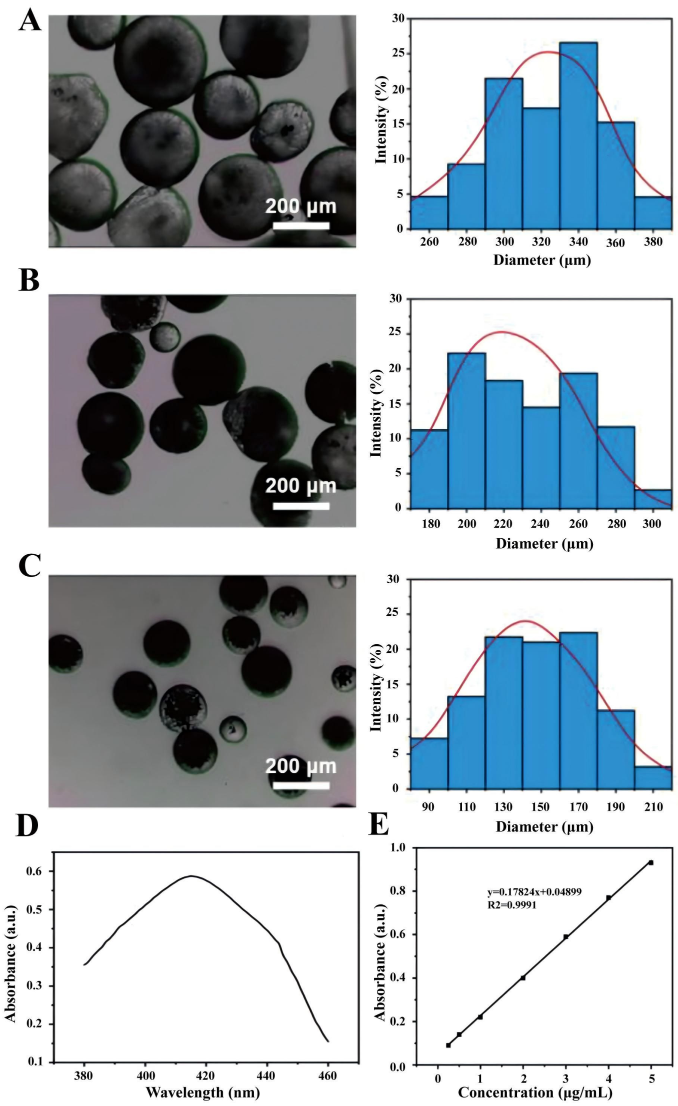

Fig S3. Effect of different stirring speeds ((A) 600, (B) 800, and (C) 1000 rpm) on microsphere size. (D) UV absorption of curcumin and (E) linear fitting equation.

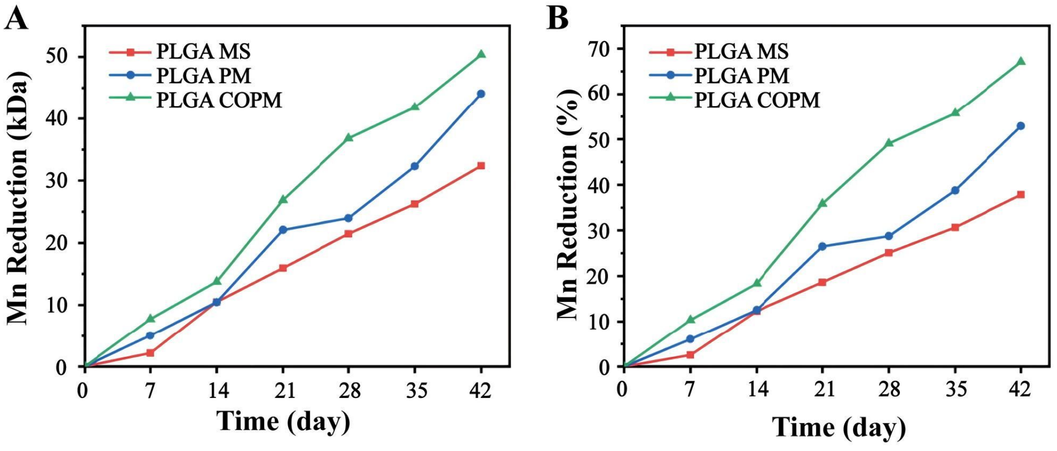

Fig S4. Changes in the molecular (A) weight reduction and (B) reduction rate during the degradation of the microspheres.

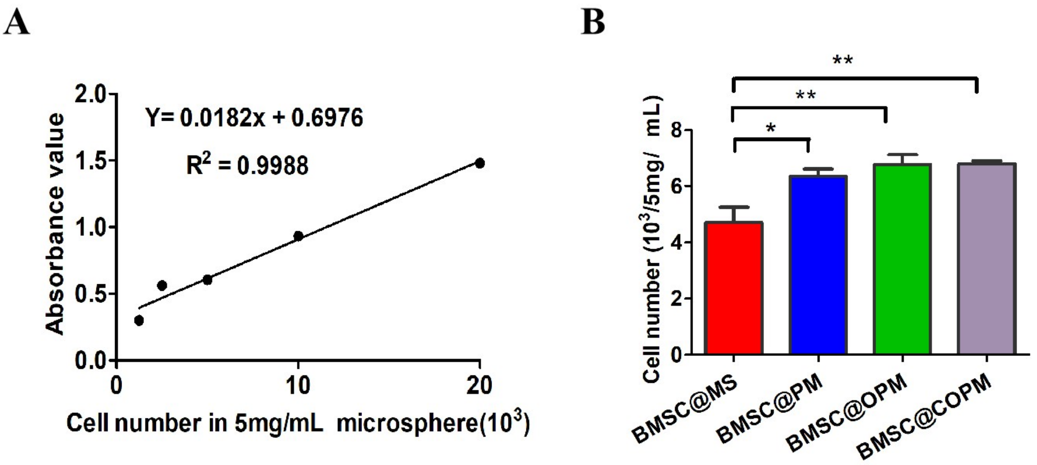

Fig S5. Loading capacity of BMSCs in microspheres. (A) Calibration curve of the relative cell number, and the absorbance was quantified via the MTT assay. (B) The relative cell number of the 5 mg/mL microsphere-treated BMSCs. Compared with the BMSC@MS group, $^{*}P < 0.05$ and $^{**}P < 0.01$; the data are presented as the means ± SDs (n = 5).

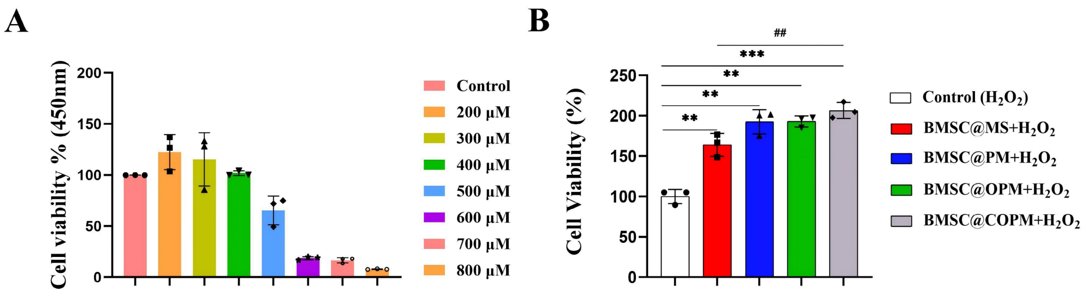

Fig S6. Screening of the H_2O_2 concentration and protection of the microspheres in a hydrogen peroxide environment (A) The concentration of hydrogen peroxide was quantified via the MTT assay. (B) Protection of BMSCs by microspheres in a 500 μM H_2O_2 environment and detection of the viability of BMSCs via the MTT assay. Compared with the control group, $^{**}P < 0.01$ and $^{***}P < 0.001$; compared with the BMSC@COPM group, $^{\#\#}P < 0.01$. The data are presented as the means ± SDs (n = 5).

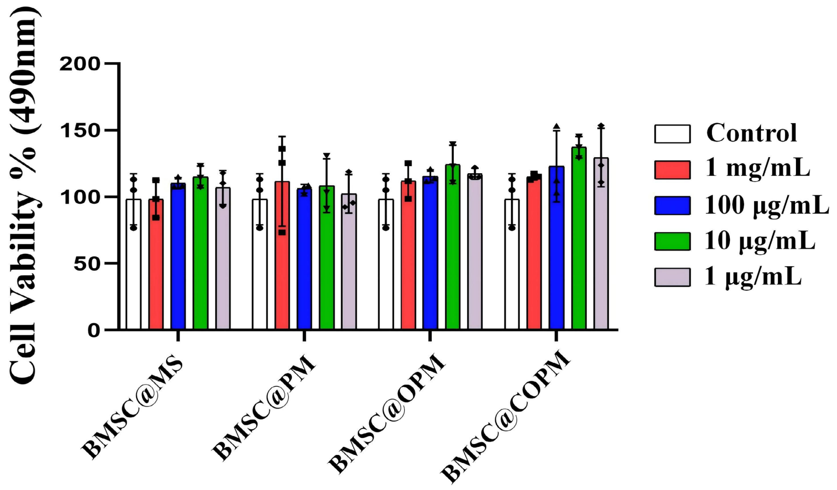

Fig S7. In vitro cytotoxicity was detected by the MTT test. The data are presented as the means ± SDs (n = 6).

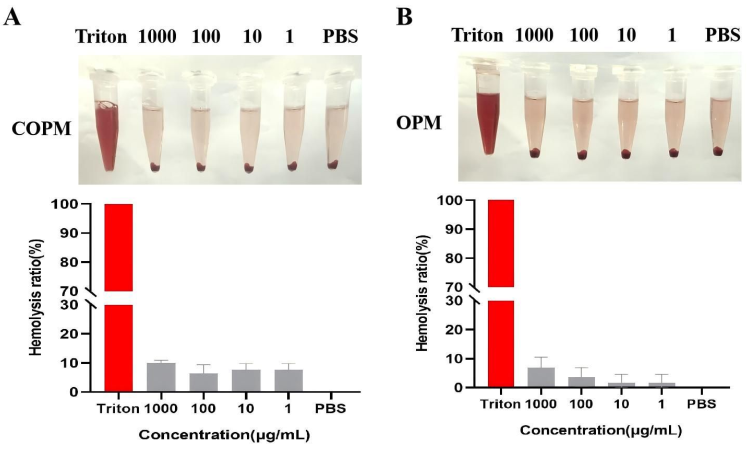

Fig S8. Representative photographs from the hemolysis assay of the COPM employing PBS as a negative control and 0.1% Triton X-100 as a positive control. (A) COPM. (B) OPM.

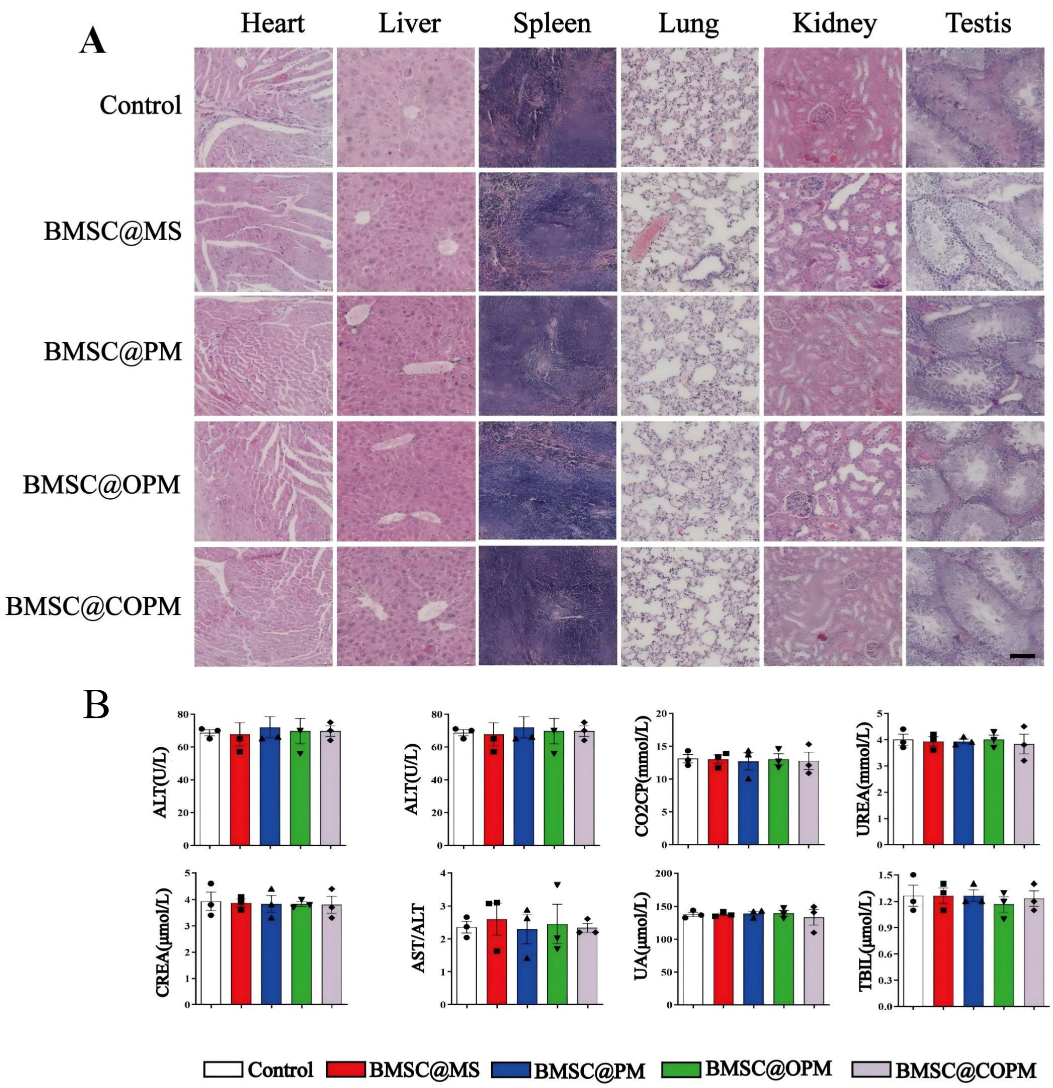

Fig S9. In vivo biocompatibility tests. (A) H&E was used to detect the internal organs of the mice (scale bar: 100 μm). (B) Blood biochemical parameters of the mice. The data are presented as the means ± SDs (n = 6).

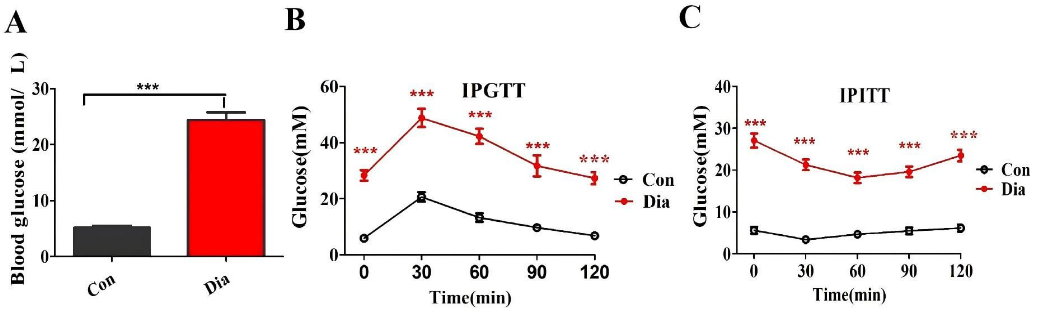

Fig S10. Results of insulin tolerance tests and glucose tolerance tests. (A) Fasting blood glucose. (B) Insulin tolerance test (IPITT) in mice. (C) Glucose tolerance test (IPGTT) in mice. The data are presented as the means ± SDs (n = 6).

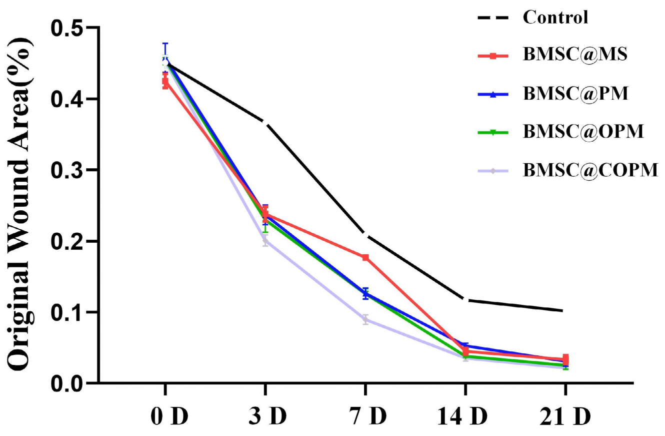

Fig S11. Statistical map of wound area changes in diabetic mice. The data are presented as the means ± SDs (n = 6).

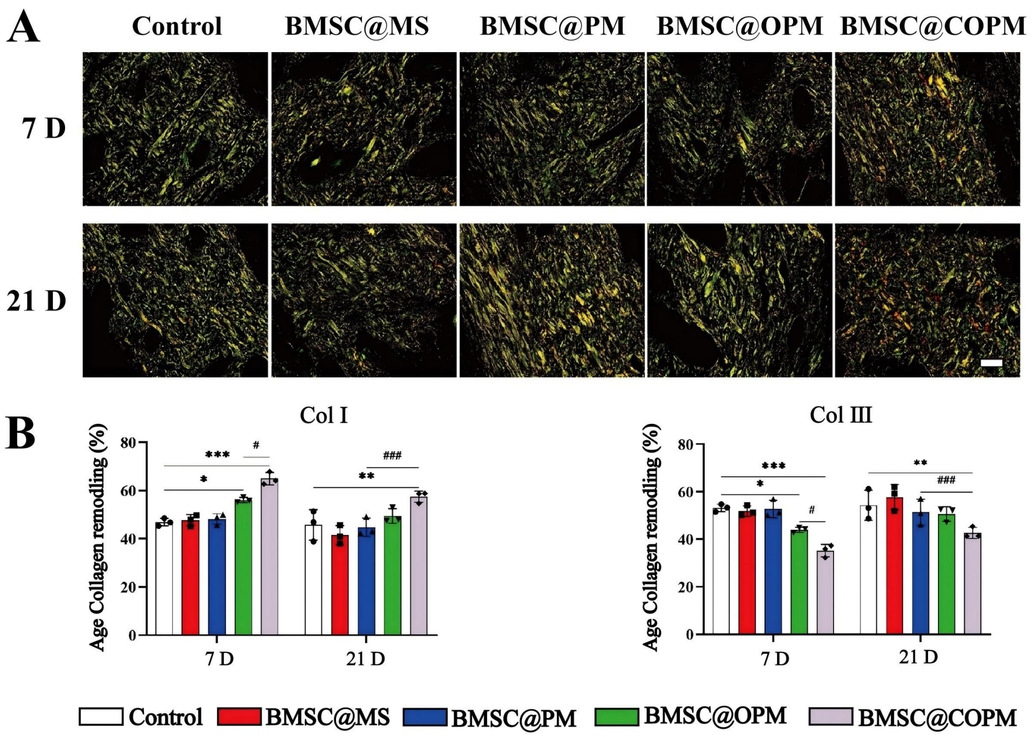

Fig S12. Sirian red staining of diabetic wounds on the 7th and 21st days. (A) Sirius red staining of diabetic wounds on the 7th and 21st days (scale bar: 100 μm). (B and C) Quantitative analysis of Sirius red S staining. Compared with the control group, $^{*}P < 0.05$, $^{**}P < 0.01$ and $^{***}P < 0.001$; compared with the BMSC@COPM group, $^{\#}P < 0.05$ and $^{\#\#\#}P < 0.001$. The data are presented as the means ± SDs (n = 3).

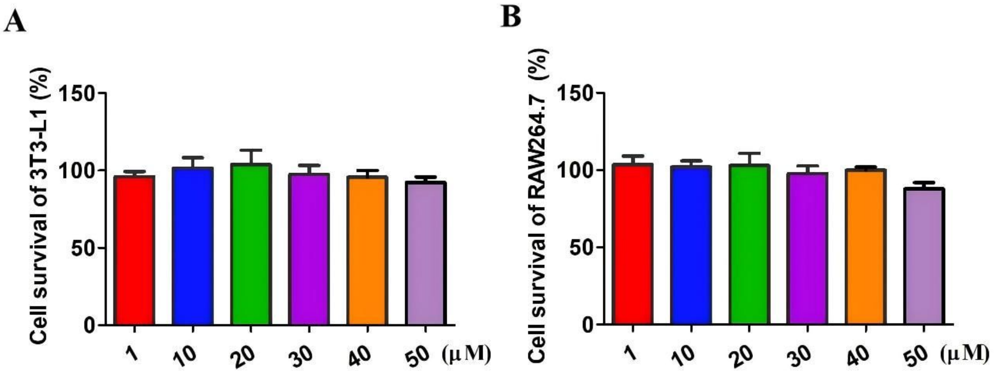

Fig S13. The effect of CQ on 3T3-L1 cells was detected by the MTT test. (A) 3T3-L1; (B) RAW264.7. The data are presented as the means ± SDs (n = 6).

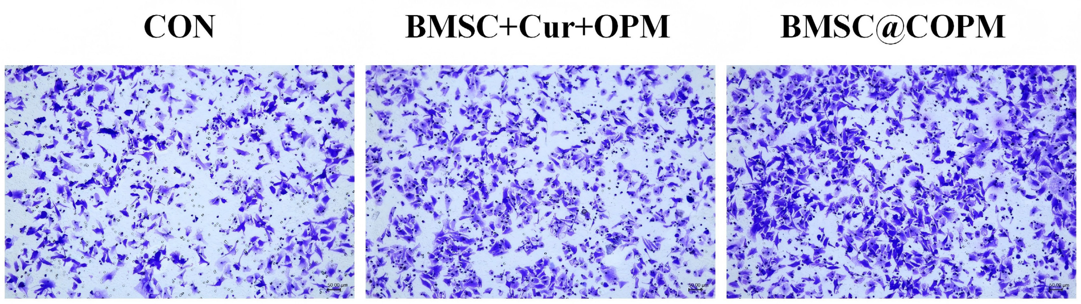

Fig S14. Compared with the simple mixture, the BMSC@COPM promoted the migration and proliferation of 3T3-L1 cells. The data are presented as the means ± SDs (n = 6).
